# Individual prognosis at diagnosis in nonmetastatic prostate cancer: Development and external validation of the PREDICT *Prostate* multivariable model

**DOI:** 10.1371/journal.pmed.1002758

**Published:** 2019-03-12

**Authors:** David R. Thurtle, David C. Greenberg, Lui S. Lee, Hong H. Huang, Paul D. Pharoah, Vincent J. Gnanapragasam

**Affiliations:** 1 Academic Urology Group, Department of Surgery, University of Cambridge, Cambridge, United Kingdom; 2 Department of Urology, Cambridge University Hospitals NHS Foundation Trust, Cambridge, United Kingdom; 3 National Cancer Registration and Analysis Service (Eastern Region), Fulbourn, Cambridge, United Kingdom; 4 Department of Urology, Singapore General Hospital, Singapore; 5 Centre for Cancer Genetic Epidemiology, Department of Oncology, University of Cambridge, Cambridge, United Kingdom; 6 Cambridge Urology Translational Research and Clinical Trials, Cambridge, United Kingdom; Peter MacCallum Cancer Centre, AUSTRALIA

## Abstract

**Background:**

Prognostic stratification is the cornerstone of management in nonmetastatic prostate cancer (PCa). However, existing prognostic models are inadequate—often using treatment outcomes rather than survival, stratifying by broad heterogeneous groups and using heavily treated cohorts. To address this unmet need, we developed an individualised prognostic model that contextualises PCa-specific mortality (PCSM) against other cause mortality, and estimates the impact of treatment on survival.

**Methods and findings:**

Using records from the United Kingdom National Cancer Registration and Analysis Service (NCRAS), data were collated for 10,089 men diagnosed with nonmetastatic PCa between 2000 and 2010 in Eastern England. Median follow-up was 9.8 years with 3,829 deaths (1,202 PCa specific). Totals of 19.8%, 14.1%, 34.6%, and 31.5% of men underwent conservative management, prostatectomy, radiotherapy (RT), and androgen deprivation monotherapy, respectively. A total of 2,546 men diagnosed in Singapore over a similar time period represented an external validation cohort. Data were randomly split 70:30 into model development and validation cohorts. Fifteen-year PCSM and non-PCa mortality (NPCM) were explored using separate multivariable Cox models within a competing risks framework. Fractional polynomials (FPs) were utilised to fit continuous variables and baseline hazards. Model accuracy was assessed by discrimination and calibration using the Harrell C-index and chi-squared goodness of fit, respectively, within both validation cohorts. A multivariable model estimating individualised 10- and 15-year survival outcomes was constructed combining age, prostate-specific antigen (PSA), histological grade, biopsy core involvement, stage, and primary treatment, which were each independent prognostic factors for PCSM, and age and comorbidity, which were prognostic for NPCM. The model demonstrated good discrimination, with a C-index of 0.84 (95% CI: 0.82–0.86) and 0.84 (95% CI: 0.80–0.87) for 15-year PCSM in the UK and Singapore validation cohorts, respectively, comparing favourably to international risk-stratification criteria. Discrimination was maintained for overall mortality, with C-index 0.77 (95% CI: 0.75–0.78) and 0.76 (95% CI: 0.73–0.78). The model was well calibrated with no significant difference between predicted and observed PCa-specific (*p* = 0.19) or overall deaths (*p* = 0.43) in the UK cohort. Key study limitations were a relatively small external validation cohort, an inability to account for delayed changes to treatment beyond 12 months, and an absence of tumour-stage subclassifications.

**Conclusions:**

‘PREDICT *Prostate’* is an individualised multivariable PCa prognostic model built from baseline diagnostic information and the first to our knowledge that models potential treatment benefits on overall survival. Prognostic power is high despite using only routinely collected clinicopathological information.

## Abstract 2

David Thurtle and colleagues present a prognostic model intended to help patients with nonmetastatic prostate cancer choose between conservative management and radical treatment.

## Author summary

**Why was this study done?:**

- Among men with nonmetastatic prostate cancer, a number of treatment options are often appropriate, including surveillance or conservative management.
- Problems of both overtreatment of indolent disease and undertreatment of aggressive disease are both recognised. Many men also suffer lifelong side effects from a treatment they may not have not needed.
- Estimating prognosis is therefore of crucial importance to inform decision-making on the benefits of treatments at the point of diagnosis. However, existing risk models are inadequate, rarely use survival as an outcome, ignore non-cancer mortality, and often group patients into broad categories. As a result, no model is yet to be formally endorsed or widely used in clinical practice.
- In this study, we sought to create an individualised model that addresses these gaps and predicts both cancer-specific and overall survival at the point of diagnosis, and that estimates the potential survival benefit of treatment.

**What did the researchers do and find?:**

- We studied a large UK dataset of over 10,000 men diagnosed with nonmetastatic prostate cancer and long-term survival information. The dataset was randomly split into model development and validation datasets. An additional dataset of over 2,500 men diagnosed in Singapore was used for additional external validation.
- Using Cox regression and fractional polynomials, models were built for 15-year prostate cancer–specific mortality and non–prostate cancer mortality using patient and tumour characteristics routinely available at diagnosis. These two models were then adjusted for competing risks to predict overall mortality.
- We found that the new risk model, called ‘PREDICT *Prostate*’, predicted survival outcomes with a high degree of accuracy in both validation sets, with concordance indices up to 0.84.
- We have now incorporated the model into a web-based interface for easy access and utility.

**What do these findings mean?:**

- To our knowledge, we present the first individualised multivariable survival model for nonmetastatic prostate cancer built and validated in an unscreened, pretreatment cohort.
- Our findings need further validation in independent datasets and may be limited by a relatively small external validation cohort.
- This tool incorporates the impact of radical therapy, which allows comparison to be made against the option of conservative management within the context of an individual’s competing risks, to inform decision-making around management.
- The model does not require any additional tests beyond standard of care, and is freely available for use. Its primary application is among men deciding between conservative management and radical treatment—where decision dilemmas are most acute.
- The model has the potential to enable well-informed and standardised decision-making and reduce both over- and undertreatment.

## Introduction

Prostate cancer (PCa) is the commonest cancer affecting males and a leading cause of cancer-related morbidity [1]. The vast majority of new presentations are with localised or locally advanced disease, representing a significant healthcare and economic burden [2]. Treatment decisions are notoriously complex, with the risk of cancer-related mortality balanced against the potential morbidity associated with treatment, as well as competing mortality risks. Estimating prognosis within these contexts is therefore highly important, with over 40,000 consultations for newly diagnosed PCa every year in the UK alone [2]. This importance has been underlined by randomised trial evidence reporting non-inferiority of conservative management compared with radical therapy in many early cancers from the American prostate cancer intervention versus observation trial (PIVOT) and the UK-based prostate testing for cancer and treatment (ProtecT) study [3,4].

Despite this importance, there are no high-quality individualised prognostic models available for clinical counselling and decision-making. Instead, tiered stratification systems are used that categorise men into different levels of risk. These models are widely endorsed by national and international guideline groups but are often derived using inadequate surrogate end points, such as prostate-specific antigen (PSA) resurgence after treatment, rather than being calibrated against mortality [5,6]. Modern extensions to these models have now sought to validate performance against cancer mortality and have extended the number of subclassifications [7–10]. Although these extensions add granularity, they remain too heterogeneous for modern individualised medicine approaches. More recent attempts at developing survival models have focussed solely on men undergoing radical treatment, and have not been appropriately validated [11,12]. The inadequacies of existing models are evident by the fact that the American Joint Committee on Cancer (AJCC) has not endorsed a single prognostic model for nonmetastatic PCa [13].

The objectives of this study were to develop and validate an individualised prognostic model for nonmetastatic PCa. Our aim was to produce a model that was able to contextualise the relative PCa-specific and overall survival outcomes for an individual with newly diagnosed disease and allow modelling of the potential benefit of treatment on these outcomes. Study design and reporting was informed by the AJCC criteria for model adoption and the transparent reporting of a multivariable prediction model for individual prognosis or diagnosis (TRIPOD) statement, respectively [14,15].

## Methods

This study is reported throughout as per the TRIPOD guideline (S1 Checklist).

### Study population and definition of variables

Fully anonymised data were retrieved from Public Health England after review by the Office for Data Release (ODR1617/171). Following approvals, Cambridge University Hospitals National Health Service Foundation Trust acted as host institution for data receipt. Information on all men diagnosed with nonmetastatic PCa in secondary care in Eastern England, UK, between 2000 and 2010 was collected prospectively by the National Cancer Registration and Analysis Service (NCRAS) Eastern Region. The cohort derivation has been previously described [16]. Men with recorded nodal or metastatic disease at diagnosis were excluded, along with men diagnosed only by endoscopic resection and any remaining men with PSA ≥ 100ng/mL, as a surrogate for occult metastatic disease [17]. Only men with intact information on key candidate predictors—age, PSA (ng/mL), histological grade group, clinical tumour stage (T-stage), and primary treatment were included. From a potential cohort of 15,335 men, 5,246 (34.2%) were excluded for missing information in at least one of these variables, leaving a final analytic cohort of 10,089. Comorbidity scores, derived from inpatient hospital episode statistics (HES) data, were also included. These are based on clinical coding of known inpatient episodes in the period between 27 and 3 months before PCa diagnosis, thus excluding PCa from any comorbidity score. Vital status was ascertained at the end of March 2017, with all analyses censored at the end of September 2016 to allow for a lag time of up to 6 months for non-cancer deaths through the National Health Service Strategic Tracing Service. Death was considered PCa specific when PCa was listed in 1a, 1b, or 1c of the death certificate.

Potential variables entered into the primary model were age, PSA, T-stage, histological grade, ethnicity, comorbidity, and primary treatment type. Information from NCRAS was that recorded at the time of diagnosis. T-stage was simplified to T1, T2, T3, or T4, as subcategories were rarely available and have limited impact in determining prognosis [18]. Histological grade groups (1–5) were used [19]. PSA (ng/mL) refers to the value at diagnosis, prior to biopsy or treatment. Primary treatment refers to the first definitive treatment the patient received in the first 12 months. Here, we have used the term conservative management to cover active surveillance and watchful waiting, as registry data did not discriminate between the two during this time period. As previously published, the majority of men receiving radiotherapy (RT) in this period were on concomitant hormone therapy, which represents current best practice for this treatment modality [20].

### Model development

The primary (UK) cohort was split randomly in a 70:30 ratio into model development (*n* = 7,062) and validation cohorts (*n* = 3,027) (Table 1). Within the development cohort, separate models were built for PCa-specific mortality (PCSM) and non-PCa mortality (NPCM). The general approach to modelling was similar to that used for the PREDICT breast cancer prognosis and treatment benefit model [21]. Cox proportional hazards models were utilised to estimate hazard ratios (HRs) associated with each candidate predictor. Follow-up time was censored at time to death, time to last follow-up, or 15 years, whichever came first. Each variable was assessed through uni- and multivariable analysis, with the proportional hazards assumption tested. A backwards elimination technique was used for variable selection with a 5% significance level. Risk relationships between continuous variables were modelled using multivariable fractional polynomials (FPs), with continuous data retained when possible to maximise predictive information. T-stage, histological grade group, and primary treatment type were modelled as factor variables. Radical treatments (RT or radical prostatectomy [RP]) were combined, as explained later. After fitting the multivariable models, smoothed functions for the baseline hazard of PCSM and NPCM were calculated. The baseline cumulative hazard was estimated for each patient, and then the logarithmic value of the baseline hazard was regressed against time using a univariate FP function [21].

**Table 1:** Baseline cohort characteristics in the UK cohort overall, model development and validation cohorts, and the external Singapore cohort.

Characteristics	Total UK Cohort		UK Model Development Cohort		UK Validation Cohort		Singapore Validation Cohort	
**Total Subjects**	10,089		7,063		3,026		2,546	
**Time at Risk (Years)**	82,887		58,138		24,750		13,416	
**Median Follow-up (Years)**	9.8	Range 0–16	9.8	Range 0–16	9.8	Range 0–16	5.1	Range 0–26
**10-Year Outcomes:**		Percent		Percent		Percent		Percent
PCa Deaths	1,030	10.2	712	10.1	317	10.5	105	4.1
Non-PCa Deaths	2,246	22.3	1555	22.0	691	22.8	225	8.8
Any-Cause Death	3,276	32.5	2,267	32.1	1,008	33.3	330	13.0
**Observations Censored before 10 Years**	3,770	37.4	2,667	37.8	1,103	36.5	1,930	75.8
**15-Year Outcomes:**								
PCa Deaths	1,202	11.9	842	11.9	360	11.9	133	5.2
Non-PCa Deaths	2,627	26.0	1,821	25.8	806	26.6	283	11.1
Any-Cause Death	3,829	38.0	2,663	37.7	1,166	38.5	416	16.3
**Observations Censored before 15 Years**	6,000	59.5	4,212	41.7	1,788	59.1	2063	81.0
**Crude PCS Mortality Rate (per Patient Year)**	1.46		1.46		1.46		0.99	
**Crude Overall Mortality Rate (per Patient Year)**	4.64		4.6		4.72		3.1	
**Age** (Mean, SD)	69.9	8.30	69.9	8.34	69.9	8.29	66.1	7.96
**PSA** (Mean, SD)	18.4	17.5	18.5	17.5	18.2	17.6	15.7	16.6
**Grade Groups**		Percent		Percent		Percent		Percent
1	3,328	33.0	2,317	32.8	1,011	33.4	1,126	44.2
2	3,017	29.9	2,125	30.1	892	29.5	723	28.4
3	1,486	14.7	1,057	15.0	429	14.2	326	12.8
4	1,032	10.2	710	10.1	322	10.6	170	6.7
5	1,226	12.2	854	12.1	372	12.3	201	7.9
**T-Stage**								
1	5,421	53.7	3,761	53.2	1,660	54.9	1,625	63.8
2	3,213	31.8	2,270	32.1	943	31.2	660	25.9
3	1,378	13.7	977	13.8	401	13.3	244	9.6
4	77	0.8	55	0.8	22	0.7	17	0.7
**Primary Treatment**								
RP	1,419	14.1	995	14.1	424	14.0	1,012	39.7
RT	3,495	34.6	2,457	34.8	1,038	34.3	823	32.3
Hormone Monotherapy	3,178	31.5	2,226	31.5	952	31.5	164	6.4
Conservative Management	1,997	19.8	1,385	19.6	612	20.2	538	21.1
Missing	na		na		na		9	0.4
**Ethnicity**								
White	7,804	77.4	5,464	77.4	2,340	77.3	36	1.4
Missing/Unknown	2,136	21.2	1,491	21.1	641	21.3	0	0.0
Asian	50	0.5	35	0.5	15	0.5	2,435	95.6
Other	99	1.0	108	1.5	26	0.9	73	2.9

Abbreviations: na, not applicable; PCa, prostate cancer; PCS, prostate cancer-specific; RP, radical prostatectomy; RT, radiotherapy; SD, standard deviation; T-stage, clinical tumour stage.

### Competing risks adjustment

Beta coefficients for each prognostic factor in the two Cox models were used to derive a prognostic index for PCSM (piPCSM) and NPCM (piNPCM) for each patient. The absolute risk (hazard(H)) of PCa death (H_PCa_) and non-PCa (H_NPC_) death until time *t*, if there were no competing mortalities, are estimated by the following formulae, respectively: H_PCa_ = 1 − exp(−exp(piPCSM) * bhPCSM(*t*)) and H_NPC_ = 1 − exp(−exp(piNPCM) * bhNPCM(*t*)), where bhPCSM(*t*) and bhNPCM(*t*) are the cumulative baseline hazards of PCSM and NPCM at time *t*, respectively. However, as these risks compete against each other, the cumulative risk (R) of overall mortality (OM) at time *t* is given by the following: R_OM_(*t*) = 1 − (1 − H_PCa_(*t*)) * (1 − H_NPC_(*t*)). Therefore, the formulae for cumulative risk (R) of PCa death and non-PCa death at time *t* are as follows: R_PCa_*(t*) = R_OM_(*t*) * (H_PCa_(*t*)/(H_PCa_(*t*) + H_NPC_(*t*)) and R_NPC_(t) = R_OM_(*t*) * (H_NPC_(*t*)/(H_NPC_(*t*) + H_PCa_(*t*)), respectively. The source code for replicating the model’s output has been made available online, including this competing risk adjustment.

### Model accuracy and comparison to existing models

Model calibration and goodness of fit was investigated in the UK validation cohort by comparing observed and predicted deaths within quintiles of predicted mortality and within strata of other prognostic variables. For assessing calibration, we integrated the predicted outcomes across all follow-up times to allow for cases with follow-up of less than 10 or 15 years. Thus, the calibration corresponds to a range of different follow-up times. A simplified χ^2^ goodness-of-fit (GOF) test was performed using the method of May and Hosmer, whereby a *p*-value of less than 0.05 would suggest a significant difference between the expected and observed number of events, assessed up to 10 years or 15 years [22]. Calibration curves were also visually assessed. Model discrimination was evaluated by estimating 10- and 15-year cumulative mortality risk. The Harrell concordance statistic (C-index) was then calculated for PCa-specific, non-PCa, and overall deaths. This accounts for right-censored data, i.e., cases with less than 10 or 15 years follow-up, respectively. All analyses were performed using Stata 14 (StataCorp, College Station, TX), with the exception of C-index, which was performed using ‘rcorr.cens’ within the ‘Hmisc’ package of R [23].

Comparisons against existing models were made by calculating C-indices for three well-known tools used at the point of diagnosis internationally—namely the University of California, San Francisco, Cancer of the Prostate Risk Assessment (CAPRA) score, the updated National Comprehensive Cancer Network (NCCN) criteria, and the three-tiered European Association of Urology (EAU) criteria [7,10,24]. Available information was used to calculate these with no imputation of missing data. Where T-stage subclassification was unknown, T-stages 2 and 3 were assumed to be T2a and T3a, respectively.

### External validation

External validation of the model was assessed using a geographically and ethnically independent cohort of men from Singapore General Hospital, diagnosed between 1990 and 2015, which has been previously described [25]. The same inclusion criteria were applied as to the model development dataset. From a potential cohort of 3,245, 699 (21.5%) were excluded for missing information. A total of 310 cases had missing data for key candidate predictors, and no follow-up was available for a further 389 men, leaving a final analysable cohort of 2,546 (Table 1). Data amongst this cohort had been recorded on a prospective basis, including the same parameters as the primary cohort, with the addition of biopsy information, but did not include comorbidity information. NPCM estimates therefore assumed the same prevalence of comorbidity as the primary dataset (10.21%), spread evenly across the cohort. Vital status was ascertained via the Singapore Ministry of Home Affairs, using the same definitions for cause of death, with data censored June 30, 2017. Model performance was assessed using the methods described above. Ethics for use of these data is covered by reference 2009/1053/D, approved by the SingHealth Centralised Institutional Review Board.

### Inclusion of biopsy information as a variable

Previous risk criteria have included diagnostic biopsy information as a potentially important prognostic variable. To investigate this, we undertook an additional sub-cohort analysis on men diagnosed at one hospital within our cohort (*n* = 1,451) for whom biopsy characteristics were available. For this, we used percentage of positive cores ([PPC] = number of cores positive for any cancer/total number of cores taken). PPC was regressed against PCSM, offset against all parameters within the base model. PPC was modelled continuously and categorically. Likelihood ratio χ^2^ tests, Akaike information criterion (AIC) and Bayesian information criterion (BIC), were used to determine best fit. The eventual parameter was weight adjusted and incorporated into the model (Tables F and G in S1 Appendix). Performance of the extended model, including the PPC parameter, was then assessed within the Singaporean cohort using the same methodology as above.

## Results

### Participants

The model development cohort consisted of 7,063 men; 842 and 1,821 men died from PCa and other causes within 15 years, respectively. The UK validation cohort consisted of 3,026 men; 360 and 806 died from PCa and other causes, respectively. Median follow-up was 9.8 years for both cohorts, with 82,887 person-years of follow-up in total (Table 1). Importantly, the UK cohort included significant numbers of patients who had undergone conservative management (*n* = 1,997). Only 114 (5.7%) of these men converted to radical treatment over total study follow-up. Trends across the inclusion period, including increased proportions of T1 disease and increasing uptake of conservative management, have been identified previously [16, 20].

### Model development and specification

**Fig 1:**
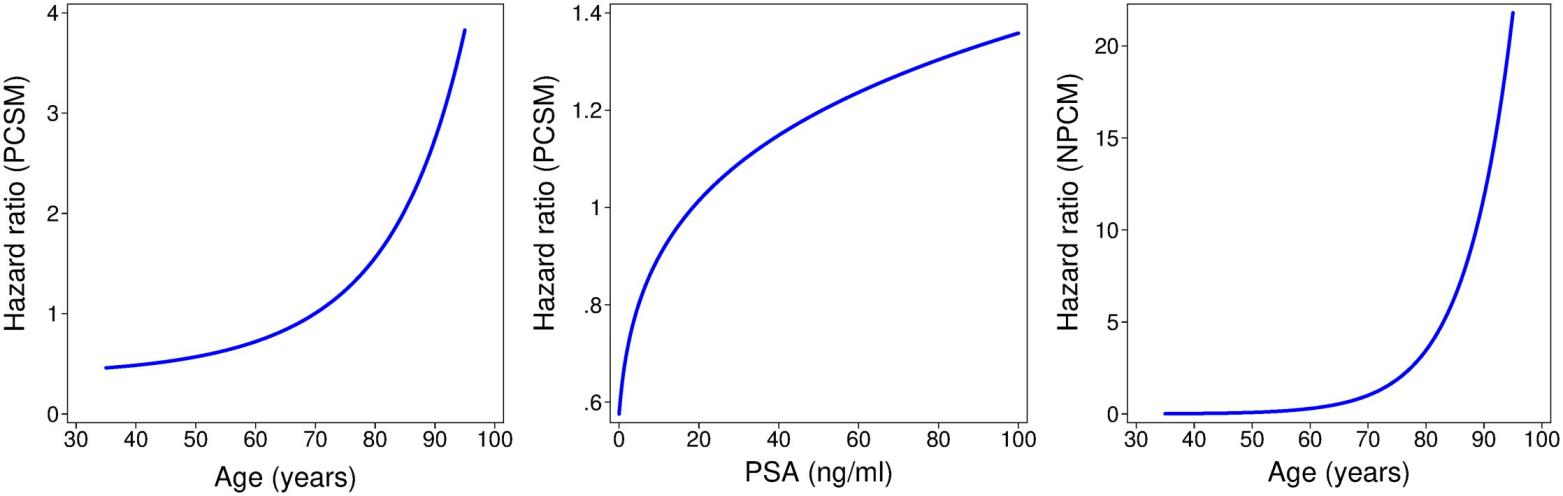
PCSM HR functions for age (left) and PSA (centre), and NPCM HR function for age (right). Each derived from the model development data. HR, hazard ratio; NPCM, non-PCa mortality; PCa, prostate cancer; PCSM, PCa-specific mortality; PSA, prostate-specific antigen.

Age, PSA, histological grade group, clinical T-stage, and primary treatment type were all independent predictors for PCSM in the development cohort (Table 2). Comorbidity had a predictive effect in relation to NPCM but not PCSM. Age was also independently prognostic for NPCM. In the final model, comorbidity was modelled as a binary variable (0 or ≥1). The HRs and FP functions for prognostic factors in the final model are shown in Table 2. Associated FP functions for age and PSA are plotted in Fig 1. These allow more flexibility in relationships for continuous variables. The estimated baseline survival functions for PCSM and NPCM are recorded in S1 Appendix and plotted against actual baseline PCSM and NPCM in Fig E in S1 Appendix.

**Table 2:** The HRs and *p*-values of the variables included in each of the PCSM and NPCM models.

Variables	PCSM		
	HR	95% CI	*p*
**Age FP**	1.003	1.002–1.003	<0.001
(age/10)^3 − 341.16			
**PSA FP**	1.204	1.092–1.328	<0.001
ln((PSA + 1)/100) + 1.6364			
**Grade Group**			
1	1.00	-	-
2	1.32	1.06–1.65	0.014
3	1.73	1.36–2.19	<0.001
4	2.10	1.63–2.69	<0.001
5	3.93	3.15–4.89	<0.001
**T-stage**			
1	1.00	-	-
2	1.18	1.01–1.37	0.042
3	1.49	1.23–1.80	0.000
4	1.88	1.14–3.13	0.014
**Primary Treatment**			
Conservative Management	1.00	-	-
Radical Treatment (RP/RT)	0.50	0.38–0.67	<0.001
Hormone Monotherapy	2.48	1.92–3.20	<0.001
	**NPCM**		
**Age FP**	1.13	1.12–1.14	<0.001
Age − 69.87			
**Comorbidity Score**			
1+	1.89	1.67–2.14	<0.001

Abbreviations: CI, confidence interval; FP, fractional polynomial; HR, hazard ratio; NPCM, non-PCa mortality; PCa, prostate cancer; PCSM, PCa-specific mortality; PSA, prostate-specific antigen; RP, radical prostatectomy; RT, radiotherapy; T-stage, clinical tumour stage.

### UK validation

**Fig 2:**
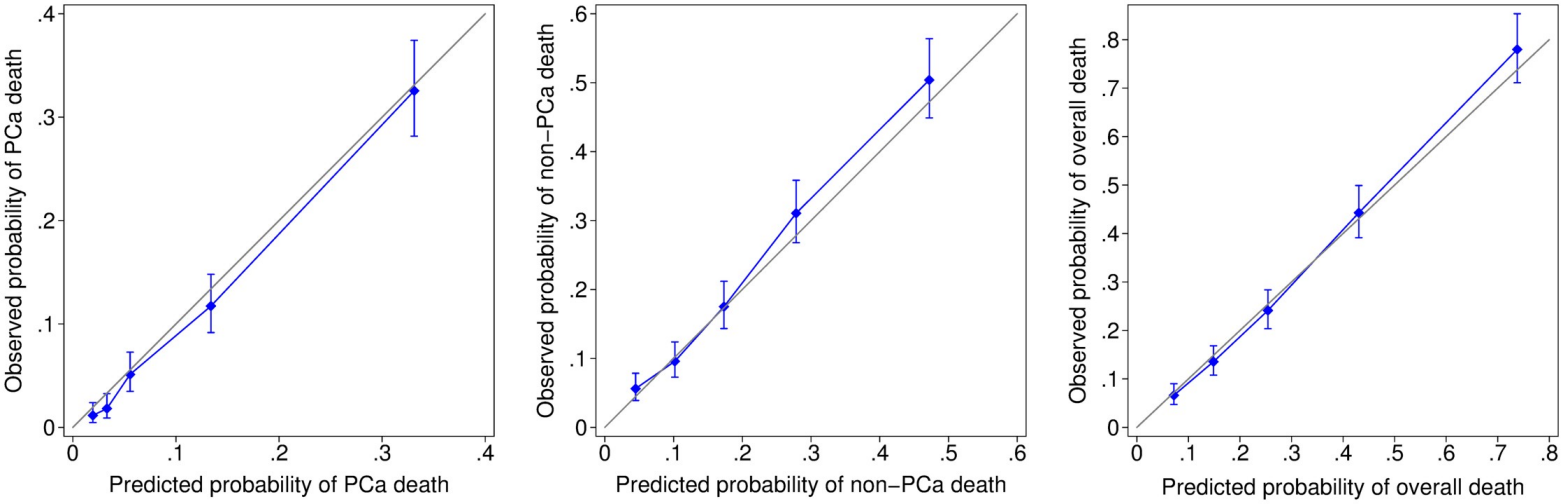
Calibration curves comparing observed and predicted probability of PCa (left), non-PCa (centre), and overall (right) deaths at 10 years, by quintile of risk, within the UK validation cohort. PCa, prostate cancer.

The model was well calibrated within the East of England validation cohort, with absolute differences between observed and predicted PCa-specific and overall deaths less than 1% at 10 years (Table 3). The GOF tests suggested the model fitted well across different quintiles of risk, as shown by the calibration curves (Fig 2), with no significant difference in observed and predicted PCa-specific (*p* = 0.19) or overall deaths (*p* = 0.43) over 10 years (Table 3). Model discrimination was good, particularly for PCSM, with C-index 0.84 (95% CI: 0.82–0.86) and 0.84 (95% CI: 0.82–0.86) over 10 and 15 years follow-up, respectively (Table 3). Within the UK cohort, model discrimination was superior (*p* < 0.001) to the current EAU, NCCN, and CAPRA risk-stratification criteria for both PCSM and overall mortality (Table 4).

**Table 3:** Observed and predicted deaths over 10 and 15 years in the UK validation cohort (*n* = 3,026). GOF and C-index are shown for each cause of death.

Follow-up duration	Predicted	Observed	Difference (%)	χ^2^ GOF	C-index	95% CI
				*p*-value		
**10-Year Events**						
PCa Deaths	343	317	−0.86	0.19	0.84	0.82–0.86
Non-PCa Deaths	641	691	1.65	0.19	0.74	0.72–0.77
Overall Deaths	986	1,008	0.73	0.43	0.77	0.75–0.78
**15-Year Events**						
PCa Deaths	413	360	−1.75	0.04	0.84	0.82–0.86
Non-PCa Deaths	751	806	1.82	0.02	0.71	0.69–0.72
Overall Deaths	1,165	1,166	0.03	0.63	0.77	0.75–0.78

Abbreviations: CI, confidence interval; GOF, goodness-of-fit; PCa, prostate cancer.

**Table 4:** Discrimination of the model, compared with other existing models amongst the UK validation cohort over 15 years maximum follow-up (*n* = 3,026).

	PCSM			Overall Mortality		
Model	C-index	95% CI	*p*	C-index	95% CI	*p*
PREDICT	0.843	0.824–0.862	-	0.766	0.753–0.780	-
EAU	0.688	0.665–0.711	<0.001	0.628	0.613–0.643	<0.001
NCCN	0.720	0.695–0.744	<0.001	0.644	0.628–0.659	<0.001
CAPRA	0.754	0.728–0.779	<0.001	0.656	0.640–0.672	<0.001

Abbreviations: CAPRA, Cancer of the Prostate Risk Assessment (UCSF); CI, confidence interval; EAU, European Association of Urology; NCCN, National Comprehensive Cancer Network; PCSM, PCa-specific mortality.

Calibration remained good across various subcategories of patients, as demonstrated in Table C in S1 Appendix. Importantly, predictions for both PCa and non-PCa deaths amongst men undergoing either conservative management or radical therapy were within 2%. The GOF tests amongst this treatment sub-cohort continued to demonstrate no significant difference between predicted and observed PCa-specific (*p* = 0.23) or overall deaths (*p* = 0.11) over 10 years.

### External validation

Accuracy of the model was also assessed using the Singaporean cohort (*n* = 2,546). Here, median follow-up was 5.1 years, with 133 and 283 PCa and non-PCa deaths, respectively (Table 1).

Model discrimination amongst this cohort was promising, with C-index 0.83 (95% CI: 0.79–0.87) and 0.76 (95% CI: 0.73–0.78) for PCSM and overall mortality, respectively, over 10 years (Table 5). Differences between observed and predicted deaths were less than 1% over 10 and 15 years, albeit within a small cohort (Table 5). GOF analysis showed no significant differences between observed and predicted non-PCa deaths, but the model appeared to slightly underestimate PCSM and overall deaths (Table 5 and Fig F in S1 Appendix). Within this external cohort, our baseline model performed better than the three tested comparators in predicting overall mortality (*p* < 0.001) (Table D in S1 Appendix). Discrimination for PCSM was improved, compared with the EAU stratification criteria, but not significantly better than the NCCN or CAPRA scores.

**Table 5:** Observed and predicted deaths over 10 and 15 years in the Singaporean validation cohort (*n* = 2,546). GOF and C-index are shown for each cause of death.

Follow-up Duration	Predicted	Observed	Difference (%)	GOF	C-index	95% CI
				*p*-value		
**10-Year Events**						
PCa Deaths	89	105	0.63	0.01	0.83	0.79–0.87
Non-PCa Deaths	236	225	−0.43	0.10	0.74	0.70–0.77
Overall Deaths	325	330	0.20	0.01	0.76	0.73–0.78
**15-Year Events**						
PCa Deaths	112	127	0.59	0.00	0.82	0.78–0.86
Non-PCa Deaths	279	273	−0.24	0.08	0.72	0.69–0.76
Overall Deaths	391	400	0.35	0.01	0.75	0.72–0.78

Abbreviations: CI, confidence interval; GOF, goodness-of-fit.

### Model extension and retesting with the inclusion of diagnostic biopsy information

After assessing multiple categorisations of PPC, PPC was integrated into the model using a dichotomous variable around a cutoff of 50% (Tables E and F in S1 Appendix). A PPC <50% or ≥50% was associated with adjusted HRs for PCSM of 0.54 and 1.78, respectively. A HR of 1.0 is applied if PPC is unknown or to omit the PPC variable (Table G in S1 Appendix).

Accuracy of the final extended model incorporating PPC was reassessed using the Singaporean cohort (*n* = 2,546). Model discrimination was slightly improved compared with the baseline model, with C-index of 0.85 (95% CI: 0.82–0.88) and 0.76 (95% CI: 0.73–0.79) for PCSM and overall mortality, respectively (Table H in S1 Appendix). Calibration was also improved with the incorporation of the PPC variable (Fig K in S1 Appendix). GOF analysis showed no significant difference between observed and predicted PCa-related deaths (*p* = 0.11), although the model still appeared to slightly underestimate PCSM. Calibration within subgroups (Table J in S1 Appendix) suggested the model underestimated PCSM in the context of very high-risk characteristics: grade group 5 (predicted: 30.6; observed: 36), T-stage 4 (predicted: 4.1; observed: 8), and PSA > 50 ng/mL (predicted: 21; observed: 25).

Next, we compared accuracy of our extended model to existing PCa models within this external cohort. The model continued to outperform existing models in predicting overall mortality (*p* < 0.001) (Table 6). For PCSM, improved C-indices were observed for PCSM compared with existing models, but again only reached significance compared with the EAU criteria. Finally, we limited the cohort to only men who received conservative management or radical treatment, to model contemporary practice, in which primary hormone therapy is less commonly used [20]. Again, the model generally showed superior discrimination compared with other models (Table K in S1 Appendix).

**Table 6:** Discrimination of the extended model, compared with other existing models amongst the Singaporean cohort over 15 years maximum follow-up (*n* = 2,546).

Model	PCSM	95% CI	*p*	Overall	95% CI	*p*
	C-index			C-index		
PREDICT	0.838	0.804–0.872	-	0.756	0.728–0.784	-
EAU	0.763	0.732–0.794	0.001	0.637	0.606–0.667	<0.001
NCCN	0.804	0.767–0.841	0.182	0.649	0.616–0.682	<0.001
CAPRA	0.822	0.785–0.860	0.530	0.671	0.638–0.704	<0.001

Abbreviations: CAPRA, (UCSF) Cancer of the Prostate Risk Assessment; CI, confidence interval; EAU, European Association of Urology; NCCN, National Comprehensive Cancer Network; PCSM, PCa-specific mortality; UCSF, University of California, San Francisco.

### Proposed clinical utility of the model

**Fig 3:**
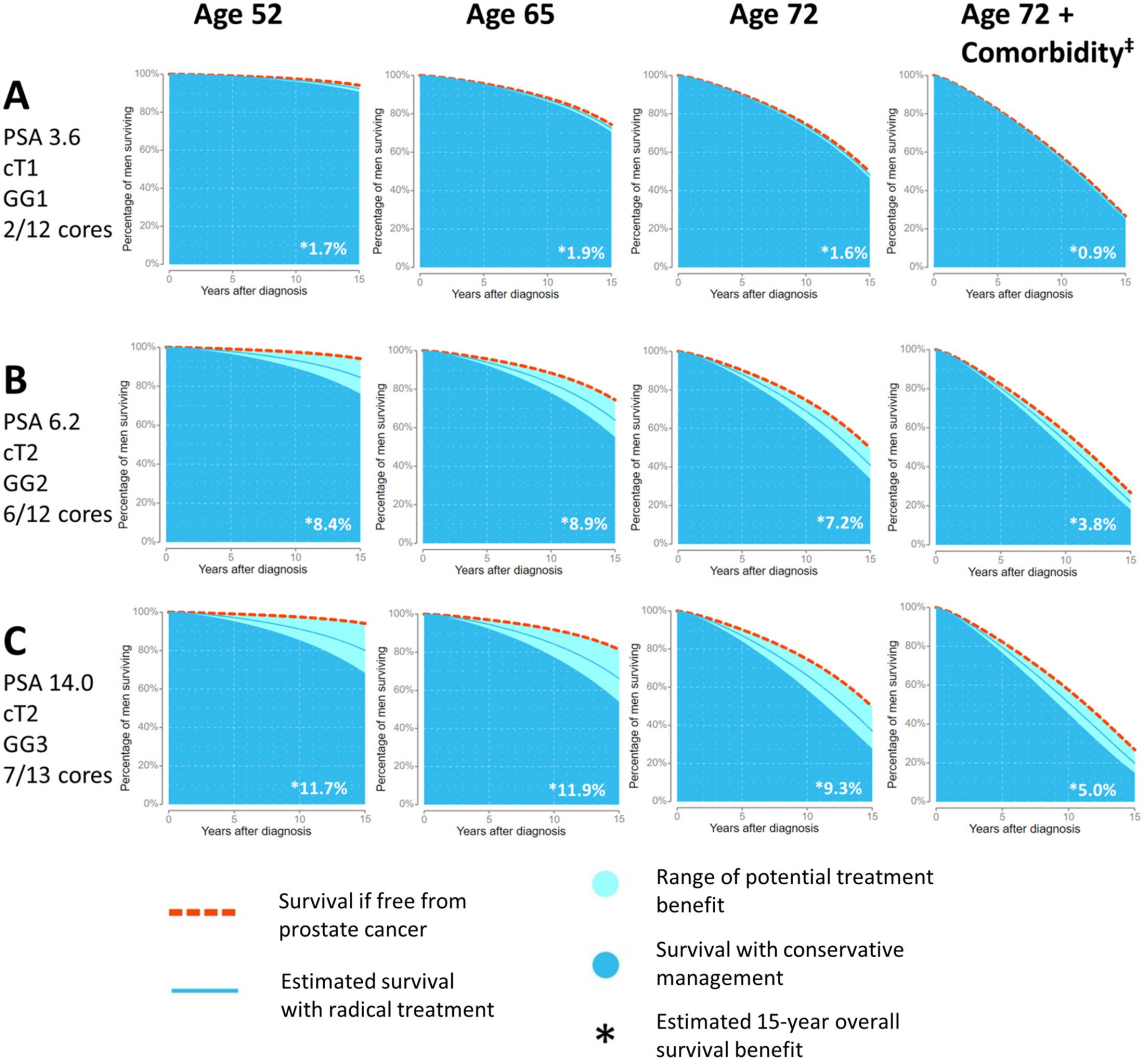
Example model outputs using 15-year overall survival curves for three hypothetical vignettes, A, B, and C. Only age and comorbidity status have been changed between each column to demonstrate the reduction in benefit from radical treatment when competing risk increases. ‡Comorbidity refers to a patient with Charlson score of 1 or more who has been admitted to hospital in the 2 years prior to PCa diagnosis. cT, clinical tumour stage; GG, histological grade group; PCa, prostate cancer; PSA, prostate-specific antigen.

To establish utility of the tool for clinicians and patients, we have developed a web-based interface for free access to the model. We expect that primary utility will be among men for whom conservative management and radical treatment might both be appropriate options. Example outputs from this web tool for three hypothetical vignettes are demonstrated in Fig 3. The age and comorbidity status at diagnosis are altered within each case to demonstrate the impact of competing risks on treatment benefit. With increasing age and comorbidity, reductions in PCSM achieved by radical treatment are attenuated by increased rates of NPCM, as the risks of PCSM and NPCM compete against one another. For example a 72-year-old with comorbidity and the disease characteristics shown in Case B has an estimated 19.6% 15-year risk of PCa death when conservatively managed. Although the estimated PCSM is reduced to 11.1% by treatment, the overall survival improves by only 3.8%, whereas for a younger man, the majority of PCSM benefit translates into overall survival benefit (Fig 3).

## Discussion

In this study, to our knowledge, we present the first individualised multivariable prognostic model for nonmetastatic PCa built and validated in an unscreened, pretreatment cohort. We show that this model, hereafter referred to as PREDICT *Prostate*, is able to derive predictions for PCa and overall mortality with a high degree of concordance by using routinely available diagnostic clinicopathological data, and appears to outperform existing models. The model incorporates the impact of radical therapy, which allows comparison to be made against the option of conservative management within the context of an individual’s competing risks. Importantly, the model does not require any additional tests or information, but could be refined in the future if additional independent factors with proven prognostic value are established.

PCa incidence is rising with an ageing male population and increased testing. In the UK alone, the incidence is projected to rise by 69% by 2030 [26]. Over 84% of UK men have nonmetastatic disease at presentation, with more than half of these classified as low or intermediate risk using traditional risk criteria [2]. Level 1 evidence shows that many men with these disease characteristics will not benefit from immediate radical therapy, with the randomised ProtecT and PIVOT trials reporting no survival differences in men managed by intervention or conservative management after 10 years of follow-up [3,4]. Additionally, radical treatment is associated with risks of significant adverse effects including incontinence, impotence, bowel dysfunction, and long-term decisional regret [27,28]. Unsurprisingly, conservative management or active surveillance is therefore becoming increasingly popular in low-risk disease, and emerging evidence also suggests very favourable outcomes in intermediate-risk disease [29].

Identifying men appropriate for initial conservative management and conveying this information to an individual within their own context of competing mortality is currently an imprecise exercise, with a lack of objective data on potential outcomes. Instead, most current prognostication is directed by categorisation of men into risk-stratified criteria and discussions with clinicians who may or may not be PCa specialists and are potentially conflicted by a bias to a treatment they offer [8–10,30]. PREDICT *Prostate* was conceived to address this critical gap in clinical need and to better inform and standardise the decision-making process. It is built around long-term actual survival data and has been designed to address all AJCC criteria [14]. The model incorporates variables available for almost any man diagnosed around the world and has wide potential applications in informing patient, clinician, and multidisciplinary team decision-making to reduce both over- and undertreatment [31]. Abundant literature shows that better decision aids contribute to more knowledgeable, informed patients and that this improves clinician-patient communication [32,33]. Therefore, we anticipate our model will be widely acceptable and highly impactful, although formal clinical impact assessments will also be undertaken [34].

The parameters used within PREDICT *Prostate* for PCSM are well-established independent variables such as grade group, PSA, and T-Stage [35–37]. Here, they have been combined in a novel way and by utilising FPs to maintain as much predictive information as possible. PREDICT *Prostate* is also distinctive in estimating the competing risks of PCSM and NPCM to accurately model overall mortality. The model deliberately uses histological grade groups (1–5) as we standardise practice towards this more intuitive scale [19]. Biopsy information was integrated as an optional variable in PREDICT *Prostate*, as biopsy quantification is accepted as a surrogate for tumour volume. However, no consensus on the best methodology for its assessment yet exists, with few studies exploring its relationship with long-term survival [38]. Hence, we used a pragmatic assessment of this by using the simplest common denominator, the number of positive versus overall biopsy cores taken (PPC). Our data showed an independent prognostic impact around the dichotomous cutoff of <50% versus ≥50% PPC. This is the same cutoff reported in two American studies exploring survival, for which effect size is comparable. This cutoff has now also been integrated into the latest NCCN risk criteria [10,39,40]. PPC thus maintains simplicity and facilitates ease of interpretation (although the model can function without biopsy information). During the study period, local practice was to perform 12-core systematic transrectal biopsy. However, contemporary practice in prostate biopsy is evolving with the use of more image targeting [41]. It is unknown how these changes will alter the prognostic value of biopsy involvement. In the meantime, we recommend adherence to the American Urological Association (AUA) guidelines, which suggest any biopsies from a target are considered as a single core if taken as part of a ‘target and systematic’ biopsy approach [9].

A key question whilst developing PREDICT *Prostate* was whether to use data-derived coefficients for treatment effect or published trial data. Ultimately, the data-derived coefficient for the combination of radical treatment types was used, with a HR of 0.50 (95% CI: 0.38–0.67). This is in fact very similar to published randomised controlled trial data of treatment effect, e.g., PIVOT (RP versus AS: HR, 0.63; 95% CI: 0.36–1.09) and ProtecT trials (RT versus active monitoring: HR, 0.51; 95% CI: 0.15–1.69. RP versus active monitoring: HR, 0.63 95% CI: 0.21–1.93) [3,4]. In the web-based presentation of the model, uncertainty around treatment effect is demonstrated by displaying treatment benefit from 0%–100% of PCSM around the estimated survival (Fig 3). Separate presentation of RT and RP outcomes was not explored, as no adequate randomised data yet shows a survival difference between the two treatment approaches [4,42]. One caveat in the clinical utility of PREDICT *Prostate* is that primary androgen deprivation, used in a proportion of our study cohorts, is now seldom used as a first-line therapy. Indeed, within this cohort, the poor prognosis apparently associated with primary androgen deprivation is likely to reflect a selection bias towards men unfit for other treatment options, or with potentially occult metastatic disease. Our model, however, is primarily for use among men deciding between conservative management and radical treatment—where decision dilemmas are most acute. Indeed, as shown in Table C in S1 Appendix, calibration of the model was best amongst men with low- to intermediate-risk features, for whom this model would be most useful and appropriate in clinical decision-making. Using disease status information from the National Prostate Cancer Audit, this may represent up to 47% of all newly diagnosed PCa [2].

Particular strengths of PREDICT *Prostate* include the derivation from a large cohort from a geographical area straddling two academic centres and nine general hospitals. These data were collected prospectively by an independent cancer registry with accurate death certificate notification, avoiding many potential biases associated with single-centre studies. The accuracy of UK PCa cause-of-death reporting is also known to be very reliable [43]. However, we do acknowledge limitations in the model. We do not have data on MRI-defined lesions or radiological stage. However, it is yet unknown if these data will improve prognostic ability, with MRI primarily used to guide biopsies rather than offer prognostic information. Indeed, the additional value of MRI in detecting missed cancers is debatable given that men with a missed cancer using non-imaging approaches have extremely low rates of PCa death [44]. The model also does not currently integrate genomic tests or molecular markers. However, the most established tools such as Prolaris CCP and Oncotype DX GPS have predominantly been tested against shorter-term outcomes in very selected groups, particularly in the posttreatment setting [45,46]. When these expensive tools have been assessed against PCSM, concordance is very similar to our model. For example, the Decipher genomic classifier alongside CAPRA showed an area under the curve (AUC) of 0.78 (95% CI: 0.68–0.87) for 10-year PCSM following prostatectomy [47]. We agree with others that good data should be sought as to whether any such marker truly adds independent prognostic information beyond a gold-standard multivariable model [48]. As with MRI, if one or more marker does show independent prognostic value in the future, it can be included in future refinements to PREDICT *Prostate* [49]. By using real world data, our treatment categories were based upon actual treatments received as opposed to assigned treatments, as is often problematic in randomised trials [4]. However, our analysis cannot account for the impact of delayed conversions to treatment beyond 1 year, albeit the number of men switching from conservative management was very small (5.7%). A final potential limitation of the model is the lack of T-stage subclassifications. However, it is accepted that T-stage is often inaccurately assigned in localised disease [18].

In terms of statistical approach, we recognise that more complex flexible parametric survival modelling frameworks exist. For example, there are several penalised regression approaches such as least absolute shrinkage and selection operator (LASSO) regression, ridge-regression, and random forests, which could have been applied. However, we have used an established methodology, which in other tumour types could not be improved upon by more complex approaches [50]. Our approach also has the advantages of allowing straightforward external validation and the incorporation of additional parameters should sufficient evidence support their inclusion, as demonstrated by updates to the PREDICT breast cancer model [51]. We also appreciate that our external validation cohort was relatively small, and different from our model-development dataset. Gaining access to well-annotated cohorts with long-term follow-up outcomes is difficult; this dataset represented the best independent cohort available to us. Applying the model in this cohort of differing case mix and ethnicity was considered a good test of the generalisability of the tool. The similar discriminatory performance herein may suggest that ethnicity is not a key determinant of prognosis. However, we recognise that follow-up duration in the Singaporean cohort is short, and the model remains untested among many other healthcare, geographic, and ethnic contexts. Finally, our comparisons to the EAU, NCCN, and CAPRA stratification criteria are pragmatic but potentially unfair. These models are intended to delineate patients into groups of risk, rather than offering predictions of 10- or 15-year risk. However, these are widely used clinical models such that these comparisons may be of interest to PCa specialists, particularly in the absence of equivalent models to compare against.

In conclusion, we have developed an individualised prognostication and decision-making tool for use at the point of PCa diagnosis. For the first time to our knowledge, this simultaneously presents individualised estimates of cancer-specific and overall survival outcomes and can model the impact of treatment on these outcomes. The accuracy of the model is promising across populations, and provides encouraging levels of discrimination in two validation cohorts. This model underpins a new web tool and decision aid to inform the decision-making process for patients and clinicians available at www.prostate.predict.nhs.uk. Further external validation of the model should be established to explore accuracy and generalisability across other contexts—particularly testing validity amongst non-Caucasians and those detected through screening.

## Supporting information

S1 Checklist TRIPOD Checklist. TRIPOD, transparent reporting of a multivariable prediction model for individual prognosis or diagnosis.
(DOCX)

S1 Proposal Prospective research proposal for doctoral project on the development and implementation of a risk prediction model for nonmetastatic PCa. PCa, prostate cancer.
(DOCX)

S1 Appendix Technical appendix to the manuscript, including additional text, tables, and figures. (DOCX)

## Abbreviations

AIC: Akaike information criterion
AJCC: American Joint Committee on Cancer
AUA: American Urological Association
AUC: area under the curve
BIC: Bayesian information criterion
CAPRA: Cancer of the Prostate Risk Assessment
EAU: European Association of Urology
FP: fractional polynomial
GOF: goodness-of-fit
HES: hospital episode statistics
HR: hazard ratio
NCCN: National Comprehensive Cancer Network
NCRAS: National Cancer Registration and Analysis Service
NPCM: non-prostate cancer mortality
PCa: prostate cancer
PCSM: PCa-specific mortality
piNPCM: prognostic index for NPCM
piPCSM: prognostic index for PCSM
PIVOT: prostate cancer intervention versus observation trial
PPC: percentage of positive cores
ProtecT: prostate testing for cancer and treatment
PSA: prostate-specific antigen
RP: radical prostatectomy
RT: radiotherapy
TRIPOD: transparent reporting of a multivariable prediction model for individual prognosis or diagnosis
T-stage: clinical tumour stage
