# Supplementary material for: Individual prognosis at diagnosis in nonmetastatic prostate cancer: Development and external validation of the PREDICT Prostate multivariable model
Source: PLoS Med. 2019 Mar 12;16(3):e1002758. doi: 10.1371/journal.pmed.1002758 (PMC6413892; doi:10.1371/journal.pmed.1002758)
Supplement: S1 Proposal — PCa, prostate cancer. (DOCX) [file pmed.1002758.s002.docx]

**MD Research Proposal:
The development and implementation of a risk prediction model for non-metastatic prostate cancer**

**David Thurtle, Paul Pharoah & Vincent Gnanapragasam**

Word count 3667

October 2016

**Introduction and significance of proposed work**

Prostate cancer (PCa) is the commonest cancer affecting males in the UK and is a leading cause of cancer-related morbidity(1). PCa incidence is increasing, with 47,300 UK men diagnosed with the disease in 2013(1). The vast majority of new presentations (>80%) are with localised or locally advanced disease(2) representing a significant healthcare and economic burden. Treatment decisions, in this growing group of men, are notoriously complex with the risk of progression and psychological impact of a cancer diagnosis balanced against significant potential morbidity associated with radical treatment options. Unlike many other conditions, there are a number of valid treatment modalities for PCa including radiotherapy, prostatectomy, androgen deprivation therapy and conservative management. Differentiating aggressive tumours that require treatment from those that are indolent, and avoiding the associated morbidity of overtreatment has been identified as the top priority in PCa research(3).

To aid the decision-making process for both clinicians and patients, risk prediction tools would be invaluable. Indeed, national UK guidelines advise that evidence-based decision aids should be used both in urological cancer multi-disciplinary teams (MDTs) and with individual patients(4). However, no useful risk prediction tool exists for UK men with non-metastatic prostate cancer. Multiple risk calculators are available, but are predominantly USA-based, built around single-centre outcome data amongst populations that are heavily radically treated and PSA-screened(5, 6). The generalisability of these models is deficient, particularly to the UK population, with a different healthcare structure and where no formal PSA screening exists. Instead, current UK guideline criteria stratify men into ‘low’, ‘intermediate’ or ‘high’ risk groups(4, 7). These groups are derived from the widely used D’Amico classification system – initially established using data from an American cohort of men to estimate the likelihood of biochemical recurrence following radical treatment(8). Biochemical recurrence has long been known to be a poor surrogate for mortality in this context(9) and many men will never undergo radical treatment, with the increasingly prevalent use of conservative management(10, 11). These three stratification groups are too broad to provide individualised predictions of outcome. However, a lack of an alternative model using high-quality data, long term follow-up and survival outcomes, necessitates the current reliance on this three stratum model.

In other tumour-types, risk prediction models are of higher quality and are already integrated in to routine clinical practice. Pioneering work in Cambridge addressed many similar themes in breast cancer, with the development of the PREDICT model(12). This web-based risk prediction tool, first published in 2010, was built using cancer registry data from the East of England. PREDICT has grown in popularity over time to become the predominant decision tool in UK breast cancer MDTs. Its website hosts approximately twenty-thousand sessions per month, with the model formally adopted by the NHS (www.predict.nhs.uk). PREDICT allows both patients and healthcare professionals to model survival outcomes, based on potential treatment modalities, and has evolved over time with the addition of contemporary biomarkers and therapeutic agents(13, 14).

There is a clear need for a novel risk prediction tool for localised prostate cancer, based upon contemporary UK data that uses mortality as an outcome measure rather than inadequate surrogates. In this proposed project, I aim to utilise the local expertise that enabled PREDICT, but adapt it for the specific context of prostate cancer. I hope to explore the prognostic variables in this cohort to create a model which can be implemented into an online, simple to use, risk prediction calculator. This will provide users with cancer-specific and overall mortality risks at diagnosis, while concurrently modelling the impacts of different treatments on these outcomes. This proposed model will be the first to be derived from a primary diagnostic cohort which is more representative of contemporary practice in the UK. It will draw from a large geographical area, with multiple healthcare institutions, and will be based upon accurate mortality data, rather than shorter term surrogate outcomes.

**Hypothesis**

We hypothesise that we can develop a validated, individualized, risk prediction tool for non-metastatic prostate cancer, that accurately stratifies men according to survival outcomes, and that this tool can be successfully integrated into clinical practice.

**Aims**

1. Establish a high-quality large contemporary database of men across East Anglia with non-metastatic prostate cancer. Use these data to develop an individualised risk prediction model that estimates overall and prostate cancer-specific survival, and the impact of different treatment modalities on these outcomes.
2. Externally validate the model and develop it in to a web-based tool for real-time clinical decision making.
3. Test the tool in urological MDTs to optimise it and assess its clinical value.

**Proposed Methods**

1. ***Development of risk model***

***Data sources***
Data will be sourced through a collaboration with Public Health England (PHE) National Cancer Registration and Analysis Service - Eastern Office. Having arranged an honorary contract at PHE, I am already assisting in the process of data extraction at the Fulbourn site to ensure the final dataset will include as many relevant variables as possible and to enable the collection of co-morbidity data using Hospital Episode Statistics (HES) data. I have met with the relevant individuals at the cancer registry who are enthusiastic about this project and eager to help.

These two data sources should complement one another by their coverage and accuracy. The cancer registry data will contain highly accurate tumour information in addition to treatment dates and endpoints of interest including mortality data. The quality of this East Anglian data is nationally recognised as being of the highest quality (15). Death certificate flagging through the Office of National Statistics ensures the registry is updated, with death notifications and vital status also regularly checked using the Health and Social Care Information Centre Personal Demographics Service batch tracing system. For cancer deaths, the lag time is a few weeks, whilst non-cancer death can take multiple months, although this lag time is being reduced. For this reason, data will be censored six months prior to the latest update of death registration.

HES is an administrative database of all inpatient hospital events in England. Recorded data includes dates of admissions, main and supplementary diagnoses, in-hospital mortality and emergency readmission data. Important information can thus be determined on clinically significant co-morbidity information for patients with known prostate cancer.

***Patient population***The study cohort will contain over 10,000 men diagnosed with localised or locally advanced non-metastatic prostate cancer between 2000 and 2010. These data are from a total population of 2.7million within the East of England region. Primary information sources include electronic and paper based reports, clinical notes and pathology results from 10 hospitals, including 2 tertiary centres. The data are therefore representative of contemporary UK clinical practice across a wide area. Dates and causes of death are maintained using additional data from the Office of National Statistics. Follow-up information and mortality data should be available until early 2016 such that median follow-up will be in the region of 10 years, significantly longer than most available UK prostate cancer cohorts.
Age, baseline PSA, clinical stage and histological score (Gleason/ISUP) are likely to be fundamental parameters to risk prediction. Missing data will be assessed and may be handled by multiple imputation if considered necessary. Patients for whom there is no co-morbidity data from HES, will still be included; and classified as having no co-morbidity.

In the past, datasets from the Anglia Cancer Network have been successfully used by colleagues in the academic urology department to demonstrate changes in the presentations and outcomes of prostate cancer over time(2, 10); thus demonstrating the feasibility of using this cancer registry data for the purpose of survival analysis. This prostate cancer cohort will be considerably larger than the cohort used to formulate the PREDICT model for breast cancer, with a similar number of cancer-specific events(12).

The primary outcome measures used for model development will be long term survival, including 10-year prostate-cancer specific mortality (PCSM) and overall mortality. Prostate-cancer specific deaths will be defined as those where prostate cancer is listed as cause 1a, 1b or 1c on the death certificate, in accordance with previous publications(16).

***Statistical analysis***Prostate cancer-specific mortality (PCSM) and non-prostate cancer mortality will be modelled separately. A Cox-proportional hazards model will be used with PCSM to create an initial model fit, with hazard ratios estimated for each prognostic factor within both a training and validation cohort. Variables will be tested in an exploratory analysis as categorical, ordinal and in the case of PSA and age, as continuous variables, to model the best fit for PCSM. The best-fit model will be derived of the observed versus predicted death rates. The effect of each treatment type will be treated as an indicator variable and the impact on cancer and non-cancer mortality outcomes evaluated.
Non-prostate cancer mortality will be modelled separately. This will likely be exponentially related to age, but also to Charlson/co-morbidity score.

Using the training cohort, a baseline survivor function for PCSM will be used to estimate the number of predicted deaths from prostate cancer at 5 and 10 years, adjusted for confirmed prognostic factors. Deaths from other causes will be estimated from baseline survivor function for other-cause mortality, adjusting for age and co-morbidity. The cumulative number of deaths in the validation cohort will be estimated by the addition of the aforementioned two estimates. Observed and predicted numbers of deaths will be compared using a Chi-squared test with further model discrimination evaluated by calculating the area under the receiver operated characteristic curve calculated for both PCSM and overall survival at 5 and 10 years from diagnosis.

Statistical software STATA™ will be used for database management and statistical processing. Statistics support will be provided through Professor Pharoah and colleagues at the Strangeways Research Laboratory. I have already undertaken basic statistical training by means of an online course in Quantitative Methods in Clinical and Public Health Research, taught by the Harvard School of Public Health. Additional statistical training, through courses run by the Medical Research Council and University College London, has also been organised.

1. **External validation and web-tool development**

This cohort, across the East of England is likely to be representative of UK practice, however, validity will need to be tested externally. Potential collaborations are being investigated with previous collaborations having taken place between co-investigators and colleagues at the Northern Ireland Cancer Registry who hold similar data fields over a similar time period. It is hoped that further cohorts can be sourced, from more ethnically-diverse, or overseas populations, to test the generalisability of the model across these populations. Dr Jem Rashbass, National Director for Disease Registration at PHE has been consulted to this end. A collaboration with colleagues in Singapore is being investigated.

External validity of the model will be tested in an appropriate external cohort. Predicted and observed death rates at 10-years will be compared, using the same methodology as that used to assess internal validity. A concordance index comparison will be applied to this independent external validation cohort.
Formal statistical comparison of this novel, validated, risk prediction tool will be performed against the current three-stratum NICE classification both within this cohort and in the external validation cohort. Co-efficients of the benefits attached to each treatment modality will be compared to previously published data.

Following external validation, hazard ratios and baseline survival data will be used to build a functioning individualised risk-prediction calculator. Individual patient and tumour characteristics at diagnosis will be entered as variables. This calculator will be built in to a web-based platform, in a similar format to the PREDICT model(13). Percentage values of overall survival will be calculated and presented in graphical format, with any incremental survival benefit of treatment modalities demonstrated. Information pages will be published on the website for both patients and professionals to explain the background, uses and disclaimers for the model.

1. **Assessment of tool in the clinical setting**

The aim of this study is not to simply develop a reliable, validated model but to produce one that is used and is beneficial in clinical practice. Although the model will be free to use, many stakeholders will expect to see evidence of clinical utility prior to adoption of the model. It is sensible to introduce the model initially to clinicians, many of whom will be familiar with the use of risk-prediction models, and who are aware of the complexities around decision-making in localised prostate cancer.

Following local approval we plan to test the web-based tool among health care professionals working in the prostate cancer field. Clinical contacts known to myself and my supervisor should enable these opportunities to assess the model’s clinical utility in both academic and general centres. Health care professionals will be informed of the background, context, validity and use of the risk prediction model. The web-based tool will then be introduced as an adjunct which can be used when discussing patients with a new diagnosis of non-metastatic prostate cancer. Initial introduction of the tool will likely be alongside hypothetical clinical vignettes, through an online survey programme. Feedback questionnaires will be requested from each participant to provide quantitative and qualitative feedback. Methodology for this purpose has been successfully used in the clinical assessment of other prostate risk assessment models which will be adapted for this setting(17). Questions will assess clinicians’ perceptions of whether the model provided additional insight in to the aggressiveness of the patient’s cancer and whether the survival outcomes were better or worse than expected. Clinicians will be asked to evaluate the ease of use and whether outputs were understandable and will be asked to hypothetically assess whether the model might have changed eventual treatment decisions.

Results will be used to amend or refine the presentation of the model and improve ease of use for clinicians. Data will be analysed to assess whether a full introduction of the model might lead to any change in decision-making or practice, in the knowledge that final treatment decisions cannot be made without patient involvement, a context in which this tool will need to be assessed separately.

**Ethical considerations**

As this is a large population-based study, with full anonymisation of data at source, ethics is not required for the initial data analysis and model construction. Any risk of using patient-identifiable information will be overcome in a number of ways. This is a large dataset, containing men with a relatively common condition. Accurate geographical information is not required, with the cohort spread over a wide area. I am working closely with Public Health England, and have met with their Caldecott Guardian, to ensure that released information is anonymised fully, yet the required information for the study is provided. For example, age at diagnosis rather than date of birth will be provided and ‘days from diagnosis to death’ rather than releasing date of death information. Permission for use of the data has already been put in writing to the Office for Data Release (ODR) at PHE.

**Pilot Data**

This project represents an extension of previous work performed in our unit in Cambridge. Data from PHE has been used previously by colleagues here to demonstrate the inadequacies of the current NICE risk stratification system, with men shown to be more accurately stratified by using 5 narrower risk groups (16). Although an updated, and more detailed dataset will be required for this particular project, from this previous study we are able to define the approximate size of the cohort (n=10,139) and be confident there will be sufficient cancer-related (>750) and overall deaths(>2500) to power our model. Although this work demonstrated the current risk categories could be improved upon by delineating tumour stage and grade more accurately, it still relied upon risk group categories and was calculated using significantly shorter follow-up data than we anticipate using. No data pertaining to co-morbidities was available in this analysis.

Cambridge is a long-term participant of the ongoing Prostate Testing for Cancer and Treatment (ProtecT) trial. The 10-year outcomes of this large multi-centre study have recently been published which demonstrated no significant survival difference between different treatment groups, including the active monitoring group(18). This study adds to the uncertainty over the best management for men with localised prostate cancer and highlights the need for a more individualised approach to assess which men may benefit most from intervention.

Over the last few months, I have spent time working at PHE on an honorary contract and have been involved in data collection. Although, I do not yet have any new analysed data, I have established good working relationships and have gained an invaluable appreciation for how the data is stored and the organisation works.

The PREDICT model in breast cancer, developed in Cambridge, has demonstrated the feasibility and clinical utility of this approach to risk prediction. Much of the methodology has been tested and refined through this local study over the last 5 years, which can be utilised in our project.

**Personal involvement**

The conception of this work is an extension of work led by Mr Gnanapragasam and others locally, looking at the changes in presentation and type of prostate cancer over time. I have a personal interest in this same topic as demonstrated by two recent publications. Firstly a paper published as joint first author in the BJUI earlier this year which explored the shifting trends of operative management for localised disease(19) and secondly in a paper recently accepted by the British Journal of Cancer demonstrating how MRI can improve risk stratification in prostate cancer(20). I have been involved in the development and refinement of the project, and coordinated the involvement of each of the collaborators through meetings and correspondence over the last year.

I am personally involved in the data extraction and database management, under the supervision of Dr Karen Wright and Dr David Greenberg at PHE. Going forward, this is likely to involve learning some SQL database code. Co-morbidity data has already been integrated with the dataset for about 10% of patients. I will perform all the database management throughout the project going forward.

I am already refreshing and building on my current knowledge of Stata™ by online study and have enrolled in upcoming statistical courses. The statistical analysis and formulation of the model will be performed myself, under the supervision of Professor Pharoah at Strangeways Research Laboratory. I hope to become proficient at statistical analysis through this project, a skill that I will be able to continue to use throughout my clinical and academic career.

Assistance will be required in the design and construction of a web-based platform for the model, although I will be closely involved to develop my information technology skills. Thereafter, the design, coordination and analysis of the piloting and testing of this model in clinical practice will be performed independently.

**Project Setting**

The work will be based across a number of sites. My work will predominantly be based at the Department of Surgery, University of Cambridge where there is dedicated office space in the Academic Urology Group Office. An honorary contract for myself is also already in place to work at the Fulbourn site of Public Health England. Here I am working on data extraction and can gain a first-hand appreciation of the quality and shortcomings of registry-based data. Data analysis, statistical input and model design will be supervised by Professor Pharoah at the Centre for Cancer Genetic Epidemiology based at Strangeways Research Laboratory, Cambridge. Here, there is access to the latest statistical software and individuals with significant experience in using and teaching statistics in biomedical research.

**Supervisors:**
Mr Vincent Gnanapragasam (University Lecturer and Honorary Consultant Urologist, Academic Urology Group, Division of Surgery, University of Cambridge).
Mr Gnanapragasam has extensive experience in all areas of urological research, with a particular interest in non-metastatic prostate cancer. He has significant experience in mentoring and supervising academic trainees and doctoral students in both basic science and clinical research. Mr Gnanapragasam has a long track record of high-impact output, much of which is in topics and studies closely aligned to the aims of this project.

Professor Paul Pharoah (Professor in cancer epidemiology, NHS Consultant in Public Health and Senior Research Group Leader, Department of Public Health & Primary Care / Department of Oncology, Centre for Cancer Genetic Epidemiology, University of Cambridge)
Professor Pharoah was one of the principal investigators involved in the creation of the PREDICT tool. He has significant experience in cancer and genetic epidemiology, with particular skills in statistical modelling and survival analyses. His recent involvement with the PREDICT model will be invaluable to the construction of the model, in addition to his skills in dissemination of results and encouraging uptake of the tool.

**Collaborators:**
Dr David Greenberg (Public Health England, National Cancer Registration and Analysis Service [Eastern Office])
Dr Greenberg has been involved in previous projects related to prostate cancer mortality. He is enthusiastic about working on this project and keen to teach me relevant skills.

**Conclusion**

I believe this project has significant potential to improve not only our knowledge surrounding best management for PCa, but also how to best relay and present this knowledge to patients. The baseline risk model could be implemented rapidly such that the project can be fast-yielding and the utility of this tool can be rapidly evaluated. We hope to not only create a statistical model, but a practical and useful clinical tool. Very importantly we will be incorporating the latest prognostic Gleason grading system(21), and using contemporary data to future-proof our tool for many years to come.  It is hoped that over the longer term, our model will allow the incorporation of novel prognostic markers and functional outcomes for ongoing refinement and improvement, such that the tool can be beneficial for both patients and researchers.

As an aspiring clinical academic this project represents the exciting opportunity of developing research, statistical and analytical skills whilst remaining close to clinical practice with clearly translational outputs. I am confident I can complete this project within the designated time permitted by the course and that whilst doing so we can produce high-quality peer-reviewed articles. It is my aim that the research conducted will be beneficial for current and future patients and that through these studies I will develop skills I can use throughout my career.

**Signature**

MD Candidate

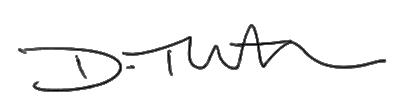


Mr D Thurtle
Academic Clinical Fellow, Urology

**Counter-signatory**
Principal Supervisor

I declare this proposal is of a standard suitable for external peer-review.


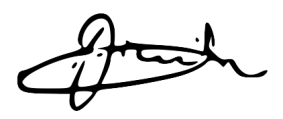


Mr VJ Gnanapragasam
University Lecturer and Honorary Consultant Urologist

1. UK Cancer Research. <http://www.cancerresearchuk.org/cancer-info/cancerstats/types/prostate/> [Available from: <http://www.cancerresearchuk.org/cancer-info/cancerstats/types/prostate/>.

2. Greenberg DC, Wright KA, Lophathanon A, Muir KR, Gnanapragasam VJ. Changing presentation of prostate cancer in a UK population--10 year trends in prostate cancer risk profiles in the East of England. Br J Cancer. 2013;109(8):2115-20.

3. JLA. James Lind Alliance Priority setting partnerships. Prostate Cancer Top 10:1. How can overtreatment for prostate cancer be prevented by identifying and excluding the treatment of harmless tumours? . 2016.

4. NICE. National Institute for Health and Care Excellence NICE Guidelines [CG175] Prostate cancer: diagnosis and treatment. January 2014.

5. MSKCC. Memorial Sloan Kettering Cancer Centre prediction tools: Prostate Cancer Nomograms. 2016.

6. Cooperberg MR, Broering JM, Carroll PR. Risk assessment for prostate cancer metastasis and mortality at the time of diagnosis. J Natl Cancer Inst. 2009;101(12):878-87.

7. Heidenreich A, Bastian PJ, Bellmunt J, Bolla M, Joniau S, van der Kwast T, et al. EAU guidelines on prostate cancer. part 1: screening, diagnosis, and local treatment with curative intent-update 2013. Eur Urol. 2014;65(1):124-37.

8. D'Amico AV, Whittington R, Malkowicz SB, Schultz D, Blank K, Broderick GA, et al. Biochemical outcome after radical prostatectomy, external beam radiation therapy, or interstitial radiation therapy for clinically localized prostate cancer. JAMA. 1998;280(11):969-74.

9. Jhaveri FM, Zippe CD, Klein EA, Kupelian PA. Biochemical failure does not predict overall survival after radical prostatectomy for localized prostate cancer: 10-year results. Urology. 1999;54(5):884-90.

10. Greenberg DC, Lophatananon A, Wright KA, Muir KR, Gnanapragasam VJ. Trends and outcome from radical therapy for primary non-metastatic prostate cancer in a UK population. PLoS One. 2015;10(3):e0119494.

11. Huland H, Graefen M. Changing Trends in Surgical Management of Prostate Cancer: The End of Overtreatment? Eur Urol. 2015.

12. Wishart GC, Azzato EM, Greenberg DC, Rashbass J, Kearins O, Lawrence G, et al. PREDICT: a new UK prognostic model that predicts survival following surgery for invasive breast cancer. Breast Cancer Res. 2010;12(1):R1.

13. Wishart GC, Bajdik CD, Dicks E, Provenzano E, Schmidt MK, Sherman M, et al. PREDICT Plus: development and validation of a prognostic model for early breast cancer that includes HER2. Br J Cancer. 2012;107(5):800-7.

14. Down SK, Lucas O, Benson JR, Wishart GC. Effect of PREDICT on chemotherapy/trastuzumab recommendations in HER2-positive patients with early-stage breast cancer. Oncol Lett. 2014;8(6):2757-61.

15. Department of Health. Delivering the cancer reform strategy. London: National Audit Office 2010 Nov 18 [cited 2016 Aug 23] [Available from: <https://www.nao.org.uk/wp-content/uploads/2010/11/1011568.pdf>.

16. Gnanapragasam VJ, Lophatananon A, Wright KA, Muir KR, Gavin A, Greenberg DC. Improving Clinical Risk Stratification at Diagnosis in Primary Prostate Cancer: A Prognostic Modelling Study. PLoS Med. 2016;13(8):e1002063.

17. Shore N, Concepcion R, Saltzstein D, Lucia MS, van Breda A, Welbourn W, et al. Clinical utility of a biopsy-based cell cycle gene expression assay in localized prostate cancer. Curr Med Res Opin. 2014;30(4):547-53.

18. Hamdy FC, Donovan JL, Lane JA, Mason M, Metcalfe C, Holding P, et al. 10-Year Outcomes after Monitoring, Surgery, or Radiotherapy for Localized Prostate Cancer. N Engl J Med. 2016.

19. Gnanapragasam VJ, Thurtle D, Srinivasan A, Volanis D, George A, Lophatananon A, et al. Evolution and oncological outcomes of a contemporary radical prostatectomy practice in a UK regional tertiary referral centre. BJU Int. 2016.

20. Thurtle D, Hsu R, Chetan M, Lophatananon A, Hubbard R, Gnanapragasam V, et al. Incorporating multi-parametric MRI-staging and the new histological Grade Group system improves risk-stratified detection of bone metastasis in prostate cancer. British Journal of Cancer (*In press*).

21. Epstein JI, Zelefsky MJ, Sjoberg DD, Nelson JB, Egevad L, Magi-Galluzzi C, et al. A Contemporary Prostate Cancer Grading System: A Validated Alternative to the Gleason Score. Eur Urol. 2016;69(3):428-35.
