# Supplementary material for: Individual prognosis at diagnosis in nonmetastatic prostate cancer: Development and external validation of the PREDICT Prostate multivariable model
Source: PLoS Med. 2019 Mar 12;16(3):e1002758. doi: 10.1371/journal.pmed.1002758 (PMC6413892; doi:10.1371/journal.pmed.1002758)
Supplement: S1 Appendix — (DOCX) [file pmed.1002758.s003.docx]

**S1 Appendix
Technical appendix to the manuscript, including additional text, tables and figures**

Individual prognosis at diagnosis in non-metastatic prostate cancer: Development and external validation of the PREDICT *Prostate* multivariable model

David R Thurtle^1,2*^, David C Greenberg^3^, Lui S Lee^4^, Hong H Huang^4^, Paul D Pharoah^5†^ & Vincent J Gnanapragasam^1,2,6†*^

1. Academic Urology Group, Department of Surgery, University of Cambridge, Cambridge, UK
2. Department of Urology, Cambridge University Hospitals NHS Foundation Trust, Cambridge, UK
3. National Cancer Registration and Analysis Service [Eastern Region], Fulbourn, Cambridge, UK
4. Department of Urology, Singapore General Hospital, Singapore
5. Centre for Cancer Genetic Epidemiology, Department of Oncology, University of Cambridge, Cambridge, UK
6. Cambridge Urology Translational Research and Clinical Trials, Cambridge, UK

**Contents**

1. Data description 3

2. Model development 6

3. UK Validation 8

4. Singapore validation of the baseline model 11

5. Model extension with the inclusion of diagnostic biopsy information 13

6. External validation of the extended model including PPC 17

7. Model comparison to existing models amongst only men on AS/RT/RP 21

1. **Data description**


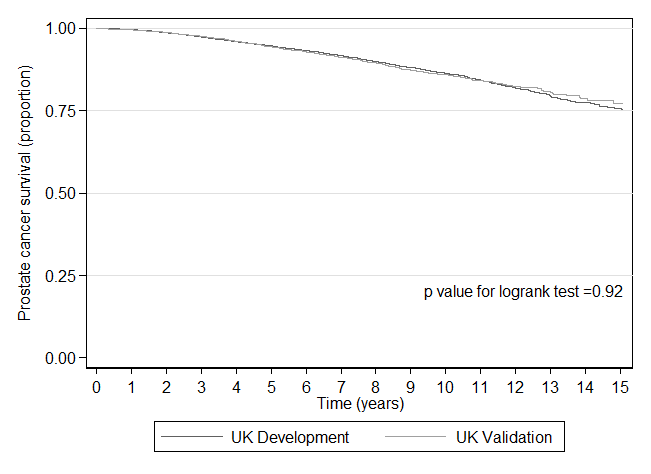

**Figure A** Non-parametric Kaplan Meier curves for prostate cancer-specific survival within the UK development and UK validation datasets.


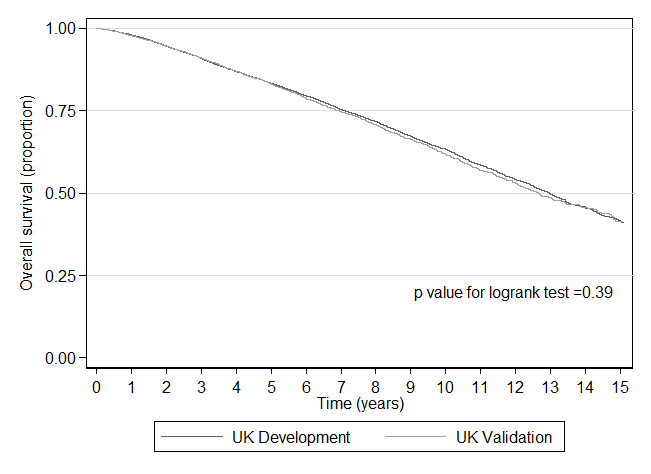


**Figure B** Non-parametric Kaplan Meier curves for overall survival within the UK development and UK validation datasets.


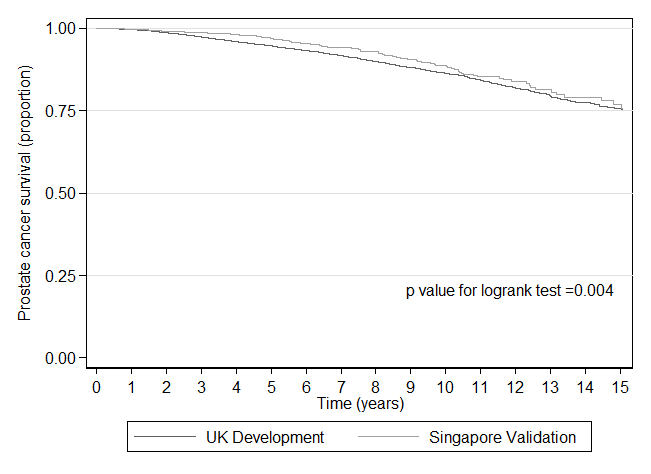

**Figure C** Non-parametric Kaplan Meier curves for prostate cancer specific survival within the UK development and Singapore validation datasets.


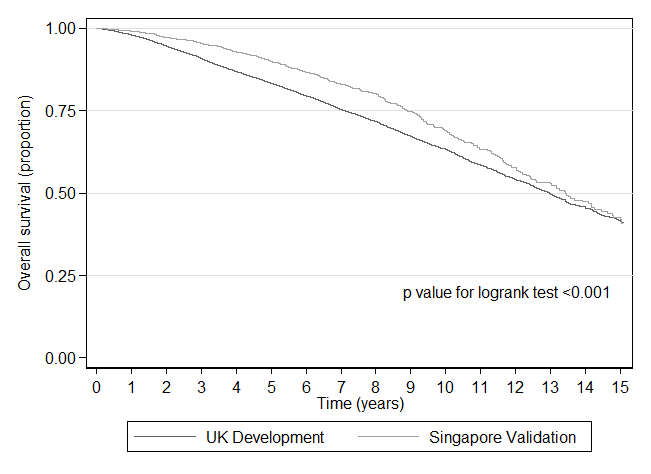


**Figure D** Non-parametric Kaplan Meier curves for prostate cancer specific survival within the UK development and Singapore validation datasets.

**2. Model Development**

**Baseline hazards**

The estimated baseline survival functions at time *t* days for PCSM and NPCM are:

Baseline hazard PCSM : exp( -16.40532 + 1.653947*(ln(*t*)) + 1.89e-12*(*t*^3)), range (0 5479)

Baseline hazard NPCM: exp(-12.4841 + 1.32274*(ln(*t*)) + 2.90e-12*(*t*^3)), range (0 5479)

These functions are plotted against actual baseline PCSM and NPCM in Figure E.


**Figure E** Baseline hazard (bh) functions for PCSM(left) and NPCM (right) plotted against observed cumulative PCSM and NPCM within the UK development cohort across 15 years.

**3. UK Validation**

|  | **Predicted** | **Observed** | **Difference (%)** | **GOF** | **C-index** | **95%CI** |
| --- | --- | --- | --- | --- | --- | --- |
|  |  |  |  | **p value** |  |  |
| **Prostate Cancer Deaths** | 343 | 317 | -0.86 | 0.19 | 0.84 | 0.82-0.86 |
| 1st quintile | 11 | 7 | -0.66 |  |  |  |
| 2nd quintile | 19 | 11 | -1.32 |  |  |  |
| 3rd quintile | 33 | 30 | -0.50 |  |  |  |
| 4th quintile | 80 | 71 | -1.49 |  |  |  |
| 5th quintile | 200 | 198 | -0.33 |  |  |  |
| **Non Prostate Cancer Deaths** | 641 | 691 | 1.65 | 0.19 | 0.74 | 0.72-0.75 |
| 1st quintile | 26 | 33 | 1.16 |  |  |  |
| 2nd quintile | 60 | 59 | -0.17 |  |  |  |
| 3rd quintile | 103 | 105 | 0.33 |  |  |  |
| 4th quintile | 167 | 188 | 3.47 |  |  |  |
| 5th quintile | 285 | 306 | 3.47 |  |  |  |
| **Overall Deaths** | 986 | 1008 | 0.73 | 0.43 | 0.77 | 0.75-0.78 |
| 1st quintile | 42 | 39 | -0.50 |  |  |  |
| 2nd quintile | 88 | 78 | -1.65 |  |  |  |
| 3rd quintile | 152 | 147 | -0.83 |  |  |  |
| 4th quintile | 258 | 270 | 1.98 |  |  |  |
| 5th quintile | 446 | 474 | 4.63 |  |  |  |

**Table A** Observed and predicted deaths across quintiles of risk within the UK validation cohort across 10 years (n=3026). χ^2^ goodness of fit (GOF) and Harrell’s C-indices are shown for each cause of death.

|  | **Predicted** | **Observed** | **Difference (%)** | **GOF** | **AUC** | **95%CI** |
| --- | --- | --- | --- | --- | --- | --- |
|  |  |  |  | **p value** |  |  |
| **Prostate Cancer Deaths** | 413 | 360 | -1.75 | 0.04 | 0.84 | 0.82-0.86 |
| 1st quintile | 13 | 6 | -1.16 |  |  |  |
| 2nd quintile | 24 | 18 | -0.99 |  |  |  |
| 3rd quintile | 43 | 42 | -0.17 |  |  |  |
| 4th quintile | 101 | 90 | -1.82 |  |  |  |
| 5th quintile | 232 | 204 | -4.63 |  |  |  |
| **Non Prostate Cancer Deaths** | 751 | 806 | 1.82 | 0.02 | 0.71 | 0.69-0.72 |
| 1st quintile | 30 | 37 | 1.16 |  |  |  |
| 2nd quintile | 72 | 76 | 0.66 |  |  |  |
| 3rd quintile | 126 | 124 | -0.33 |  |  |  |
| 4th quintile | 201 | 191 | -1.65 |  |  |  |
| 5th quintile | 322 | 378 | 9.26 |  |  |  |
| **Overall Deaths** | 1165 | 1166 | 0.03 | 0.63 | 0.77 | 0.75-0.78 |
| 1st quintile | 48 | 46 | -0.33 |  |  |  |
| 2nd quintile | 105 | 97 | -1.32 |  |  |  |
| 3rd quintile | 187 | 176 | -1.82 |  |  |  |
| 4th quintile | 316 | 313 | -0.50 |  |  |  |
| 5th quintile | 509 | 534 | 4.13 |  |  |  |

**Table B** Observed and predicted deaths across quintiles of risk within the UK validation cohort across 15 years (n=3026). χ^2^ goodness of fit (GOF) and Harrell’s C-indices are shown for each cause of death.

| **Category** | Number of cases | **PCa deaths** | |  | **NPCa Deaths** | |  | **Overall deaths** | |  |
| --- | --- | --- | --- | --- | --- | --- | --- | --- | --- | --- |
| **Age at diagnosis** |  | Predicted | Observed | Diff. (%) | Predicted | Observed | Diff. (%) | Predicted | Observed | Diff. (%) |
| <60 | 317 | 10.9 | 11 | 0.0 | 10.7 | 12 | 0.4 | 21.6 | 23 | 0.4 |
| 60-69 | 1,121 | 68.8 | 72 | 0.3 | 118.8 | 131 | 1.1 | 187.7 | 203 | 1.4 |
| 70-79 | 1,207 | 166.1 | 152 | -1.2 | 329.1 | 342 | 1.1 | 495.2 | 494 | -0.1 |
| ≥80 | 381 | 100.9 | 82 | -5.0 | 189.1 | 206 | 4.4 | 290.0 | 288 | -0.5 |
| **PSA (ng/ml)** |  |  |  |  |  |  |  |  |  |  |
| 0<10 | 1,176 | 68.6 | 56 | -1.1 | 180.5 | 166 | -1.2 | 249.1 | 222 | -2.3 |
| 10<20 | 1,025 | 106.6 | 90 | -1.6 | 237.7 | 255 | 1.7 | 344.3 | 345 | 0.1 |
| 20<50 | 597 | 111.5 | 106 | -0.9 | 168.5 | 207 | 6.4 | 280.0 | 313 | 5.5 |
| ≥50 | 228 | 60.1 | 65 | 2.1 | 61.0 | 63 | 0.9 | 121.0 | 128 | 3.1 |
| **T Stage** |  |  |  |  |  |  |  |  |  |  |
| 1 | 1,660 | 160.8 | 143 | -1.1 | 366.7 | 389 | 1.3 | 527.4 | 532 | 0.3 |
| 2 | 943 | 115.9 | 94 | -2.3 | 202.7 | 222 | 2.0 | 318.6 | 316 | -0.3 |
| 3 | 401 | 63.0 | 70 | 1.7 | 72.9 | 75 | 0.5 | 136.0 | 145 | 2.2 |
| 4 | 22 | 7.0 | 10 | 13.6 | 5.4 | 5 | -1.8 | 12.4 | 15 | 11.8 |
| **Grade Group** |  |  |  |  |  |  |  |  |  |  |
| 1 | 1,011 | 63.3 | 61 | -0.2 | 206.9 | 216 | 0.9 | 270.1 | 277 | 0.7 |
| 2 | 892 | 62.6 | 33 | -3.3 | 165.5 | 163 | -0.3 | 228.1 | 196 | -3.6 |
| 3 | 429 | 55.8 | 45 | -2.5 | 102.3 | 99 | -0.8 | 158.1 | 144 | -3.3 |
| 4 | 322 | 58.9 | 53 | -1.8 | 84.2 | 108 | 7.4 | 143.1 | 161 | 5.6 |
| 5 | 372 | 106.2 | 125 | 5.1 | 88.8 | 105 | 4.4 | 195.0 | 230 | 9.4 |
| **Primary Treatment** |  |  |  |  |  |  |  |  |  |  |
| Conservative Management | 612 | 36.8 | 28 | -1.4 | 148.7 | 144 | -0.8 | 185.5 | 172 | -2.2 |
| RT/RP | 1,462 | 57.7 | 51 | -0.5 | 204.3 | 177 | -1.9 | 262.0 | 228 | -2.3 |
| Hormone monotherapy | 952 | 252.2 | 238 | -1.5 | 294.7 | 370 | 7.9 | 546.9 | 608 | 6.4 |
| **Comorbidity** |  |  |  |  |  |  |  |  |  |  |
| Nil | 2,696 | 299.8 | 278 | -0.8 | 527.0 | 563 | 1.3 | 826.7 | 841 | 0.5 |
| ≥1 | 330 | 47.0 | 39 | -2.4 | 120.7 | 128 | 2.2 | 167.8 | 167 | -0.2 |

**Table C** Calibration between observed and predicted PCSM, NPCM and overall mortality at 10 years for sub-groups within the UK validation cohort.

**4. Singapore validation of the baseline model**

**
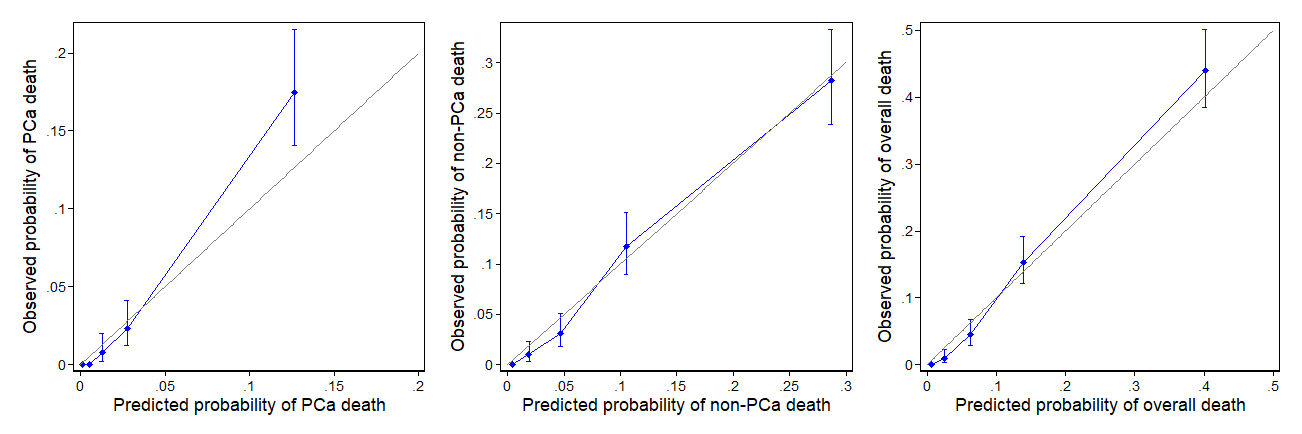
**

**Figure F** Calibration curves comparing observed and predicted prostate cancer (PCa) (left), non-PCa (centre) and overall (right) deaths at 10 years by quintile of risk using the baseline model within the Singapore validation cohort.

|  | **PCSM** |  |  | **Overall** |  |  |
| --- | --- | --- | --- | --- | --- | --- |
| Model | C-index | 95% CI | p | C-index | 95% CI | p |
| PREDICT | 0.822 | 0.780-0.864 | - | 0.749 | 0.721-0.777 | - |
| EAU | 0.763 | 0.732-0.794 | 0.026 | 0.6365 | 0.606-0.667 | <0.001 |
| NCCN | 0.804 | 0.772-0.846 | 0.529 | 0.6587 | 0.627-0.690 | <0.001 |
| CAPRA | 0.822 | 0.785-0.860 | 1.01 | 0.67079 | 0.638-0.704 | <0.001 |

**Table D** Discrimination of the model, compared to other existing models amongst the Singapore validation cohort over 15 years maximum follow-up (n=3026).
EAU = European Association of Urology NCCN = National Comprehensive Cancer Network CAPRA = UCSF Cancer of the Prostate Risk Assessment

**5. Model extension with the inclusion of diagnostic biopsy information**

To explore the value of biopsy core involvement as a prognostic factor we analysed a sub-cohort of 1,451 men diagnosed at a single centre for whom percentage positive cores (PPC) information was available. The unadjusted rates of PCSM within ten categories of PPC in this sub-cohort are shown in Figure G. A step-change in poorer prognosis was observed with PPC ≥50% (Figure G), as is also demonstrated by the relative hazard ratios for PCSM for categories above 50% (Figure H). PPC was also modelled using several different categorisations, including using these data continuously. However, PPC using a dichotomous variable around a cut-off of 50% best fit these data as demonstrated in Table E and Figure I, with the differences in survival also demonstrated in Figure J.

Nine hundred and thirty-nine (63.0%) and 552 (37.0%) men had <50% and ≥50% PPC respectively. The hazard ratio (HR) for PCSM was 3.31 for those with ≥50% PPC compared to 1.0 for those with PPC <50% (Table F). However, to incorporate PPC into the model these hazard ratios required adjustment for the relative proportions of the two groups, such that the net effect remains a hazard of 1.0. Therefore the ‘biopsy effect’ is only activated if PPC is known. If PPC is unknown, predictions are as per the baseline model. Core involvement was weight-adjusted according to these relative proportions of PPC, as shown in Table F: First the HR is multiplied by the proportion to calculate the ‘weighting’. Next, the ‘adjusted HR’ is calculated by dividing the HR by the sum of the two weightings. The coefficients, for inclusion in the model, are calculated by taking the log of the adjusted HR, in the usual way. The final composition of the ‘extended model’ including PPC is shown in Table G.


**Figure G** Proportion of men dying from prostate cancer within each decile of percentage positive cores (PPC), without any adjustment for other parameters.

**Figure H** Hazard ratios for PCSM within deciles of percentage positive cores (PPC), following full adjustment for parameters included in the model.

**Figure I** Likelihood-Ratio Chi squared values assessing every potential value of PPC as a cut-off for a dichotomous variable. The highest figures were for ≥48, ≥49 or ≥50% PPC (highlighted red).

| **Categorisation** | **AIC** | **BIC** |
| --- | --- | --- |
| PPC (continuous) | 1160.0 | 1165.3 |
| Logit transformation of PPC | 1162.3 | 1167.6 |
| <50% vs ≥50% | 1154.8 | 1160.0 |
| <50% vs 50<75% vs 75-100% | 1156.5 | 1167.1 |
| <33% vs 33-67% vs 67-100% | 1318.2 | 1328.9 |

**Table E** Akaike (AIC) and Bayesian information criterion (BIC) for various categorisations of PPC.

| PPC | Unadjusted HR | Frequency | Proportion | Weighting (HR*proportion) | | Weight-adjusted HR |
| --- | --- | --- | --- | --- | --- | --- |
| <50% | 1 | 939 | 0.63 | 0.630 | **0.54** | |
| ≥50% | 3.31 | 552 | 0.37 | 1.225 | **1.78** | |
|  |  |  |  | Sum: 1.855 |  | |

**Table F** The unadjusted hazard ratios (HR) for prostate cancer specific mortality (PCSM) for the two PPC categories are shown. These are adjusted according to the proportion of men in each PPC category to provide a weight-adjusted HR.


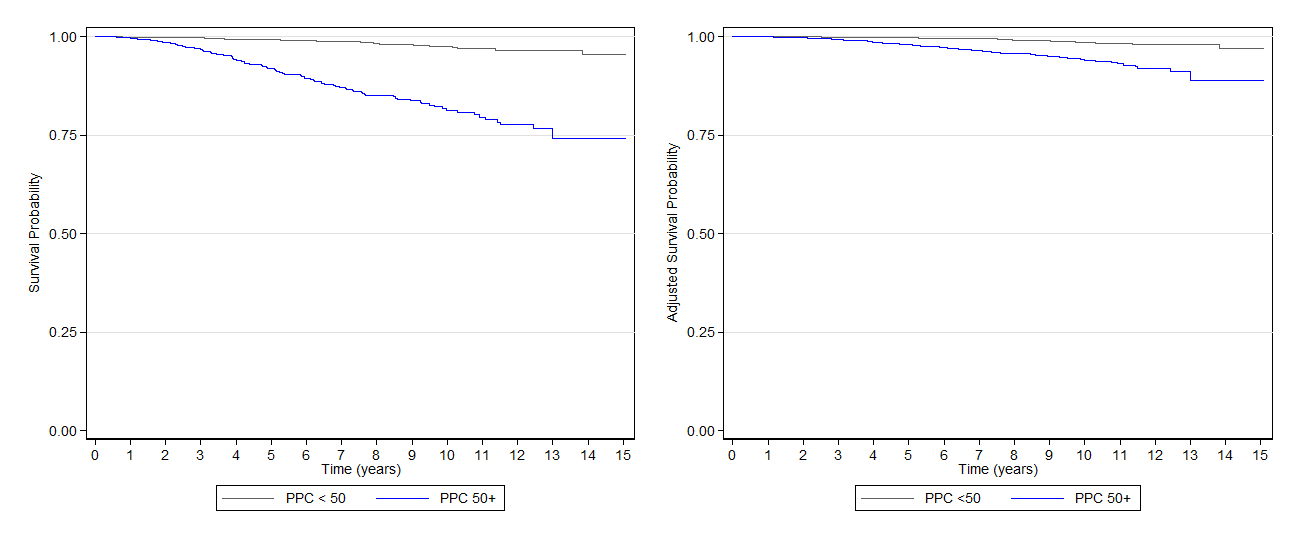


**Figure J** Kaplan Meier curves showing the differences in survival for men with percentage positive biopsy cores (PPC) of 50% or greater compared to those with less than 50% PPC. The left graph is unadjusted, whereas the right graph shows the curve adjusted for all other prognostic variables for prostate cancer specific mortality within the multi-variable model.

|  | **Prostate Cancer Specific Mortality** | | |
| --- | --- | --- | --- |
|  | HR | 95%CI | P |
| **Age FP** | 1.003 | 1.002-1.003 | <0.001 |
| (age/10)^3 -341.16 |  |  |  |
| **PSA FP** | 1.204 | 1.092-1.328 | <0.001 |
| ln((psa+1)/100)+1.6364 |  |  |  |
| **Grade group** |  |  |  |
| **1** | 1.00 | - | - |
| **2** | 1.32 | 1.06-1.65 | 0.014 |
| **3** | 1.73 | 1.36-2.19 | <0.001 |
| **4** | 2.10 | 1.63-2.69 | <0.001 |
| **5** | 3.93 | 3.15-4.89 | <0.001 |
| **T stage** |  |  |  |
| **1** | 1.00 | - | - |
| **2** | 1.18 | 1.01-1.37 | 0.042 |
| **3** | 1.49 | 1.23-1.80 | 0.000 |
| **4** | 1.88 | 1.14-3.13 | 0.014 |
| **Percentage positive cores (PPC)** |  |  |  |
| **<50%** | 0.54 | -- | <0.001 |
| **Unknown** | 1.00 |  |  |
| **≥50%** | 1.78 | -- | <0.001 |
| **Primary Treatment** |  |  |  |
| Conservative management | 1.00 | - | - |
| Radical treatment (RP/RT) | 0.50 | 0.38-0.67 | <0.001 |
| Hormone monotherapy | 2.48 | 1.92-3.20 | <0.001 |
|  | **Non Prostate Cancer Mortality** | | |
| **Age FP** | 1.13 | 1.12-1.14 | <0.001 |
| age-69.87 |  |  |  |
| **Comorbidity Score** |  |  |  |
| **1+** | 1.89 | 1.67-2.14 | <0.001 |

**Table G** The hazard ratios and p values of the variables included in each of the prostate cancer specific mortality and non-prostate cancer mortality models, including the incorporation of the percentage positive cores (PPC) variable. FP = fractional polynomial HR = hazard ratio CI = confidence interval

**6. External validation of the extended model including PPC**


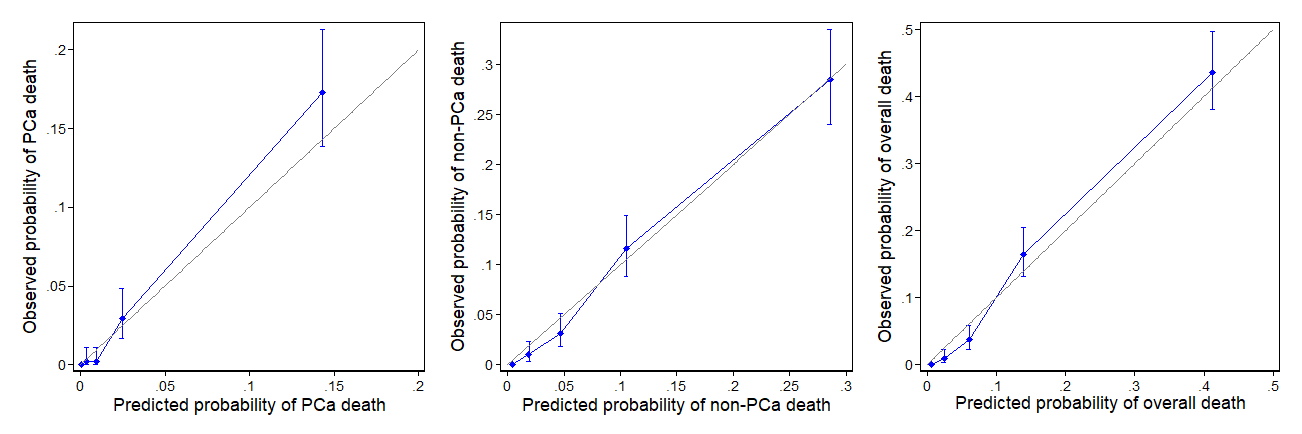


**Figure K** Calibration curves comparing observed and predicted prostate cancer (PCa) (left), non-PCa (centre) and overall (right) deaths at 10 years by quintile of risk amongst the Singapore validation dataset, using the extended model including PPC.

|  | **Predicted** | **Actual** | **Difference (%)** | **GOF** | **C-index** | **95%CI** |
| --- | --- | --- | --- | --- | --- | --- |
|  |  |  |  | **p value** |  |  |
| **Prostate Cancer Deaths** | 92 | 105 | 0.51 | 0.11 | 0.85 | 0.82-0.88 |
| 1st quintile | 0 | 0 | 0.00 |  |  |  |
| 2nd quintile | 2 | 1 | -0.20 |  |  |  |
| 3rd quintile | 5 | 1 | -0.79 |  |  |  |
| 4th quintile | 12 | 15 | 0.59 |  |  |  |
| 5th quintile | 73 | 88 | 2.95 |  |  |  |
| **Non Prostate Cancer Deaths** | 236 | 225 | -0.43 | 0.23 | 0.74 | 0.70-0.77 |
| 1st quintile | 2 | 0 | -0.39 |  |  |  |
| 2nd quintile | 10 | 5 | -0.98 |  |  |  |
| 3rd quintile | 24 | 16 | -1.57 |  |  |  |
| 4th quintile | 54 | 59 | 0.98 |  |  |  |
| 5th quintile | 145 | 145 | 0.00 |  |  |  |
| **Overall Deaths** | 328 | 330 | 0.08 | 0.00 | 0.76 | 0.73-0.79 |
| 1st quintile | 3 | 0 | -0.59 |  |  |  |
| 2nd quintile | 13 | 5 | -1.57 |  |  |  |
| 3rd quintile | 31 | 19 | -2.36 |  |  |  |
| 4th quintile | 71 | 84 | 2.55 |  |  |  |
| 5th quintile | 210 | 222 | 2.36 |  |  |  |

**Table H** Observed and predicted deaths across quintiles of risk using the extended model including PPC within the Singapore validation cohort across 10 years (n=2546). Goodness of fit (GOF) and Harrell’s C-indices are shown for each cause of death.

|  | **Predicted** | **Actual** | **Difference (%)** | **GOF** | **Harrell’s**  **C** | **95%CI** |
| --- | --- | --- | --- | --- | --- | --- |
|  |  |  |  | **p value** |  |  |
| **Prostate Cancer Deaths** | 114 | 127 | 0.51 | 0.08 | 0.84 | 0.80-0.87 |
| 1st quintile | 0 | 0 | 0.00 |  |  |  |
| 2nd quintile | 2 | 1 | -0.20 |  |  |  |
| 3rd quintile | 5 | 1 | -0.79 |  |  |  |
| 4th quintile | 15 | 13 | -0.39 |  |  |  |
| 5th quintile | 92 | 112 | 3.93 |  |  |  |
| **Non Prostate Cancer Deaths** | 278 | 273 | -0.20 | 0.17 | 0.72 | 0.68-0.75 |
| 1st quintile | 2 | 0 | -0.39 |  |  |  |
| 2nd quintile | 10 | 4 | -1.18 |  |  |  |
| 3rd quintile | 25 | 18 | -1.38 |  |  |  |
| 4th quintile | 60 | 67 | 1.38 |  |  |  |
| 5th quintile | 181 | 184 | 0.59 |  |  |  |
| **Overall Deaths** | 393 | 400 | 0.27 | 0.00 | 0.76 | 0.73-0.78 |
| 1st quintile | 3 | 0 | -0.59 |  |  |  |
| 2nd quintile | 13 | 5 | -1.57 |  |  |  |
| 3rd quintile | 32 | 17 | -2.95 |  |  |  |
| 4th quintile | 81 | 102 | 4.13 |  |  |  |
| 5th quintile | 264 | 276 | 2.36 |  |  |  |

**Table I** Observed and predicted deaths across quintiles of risk using the extended model including PPC within the Singapore validation cohort across 15 years (n=2546). Goodness of fit (GOF) and Harrell’s C-indices are shown for each cause of death.

| **Category** | Number of cases | **Prostate cancer deaths** | |  | **Non prostate cancer deaths** | | | **Overall deaths** | |  |
| --- | --- | --- | --- | --- | --- | --- | --- | --- | --- | --- |
| **Age at diagnosis** |  | Predicted | Observed | Diff. (%) | Predicted | Observed | Diff. (%) | Predicted | Observed | Diff. (%) |
| <60 | 501 | 5.8 | 10 | 0.8 | 8.0 | 10 | 0.4 | 13.8 | 20 | 1.2 |
| 60-69 | 1,196 | 27.0 | 31 | 0.3 | 65.5 | 66 | 0.0 | 92.5 | 97 | 0.4 |
| 70-79 | 737 | 39.9 | 46 | 0.8 | 116.3 | 106 | -1.4 | 156.3 | 152 | -0.6 |
| ≥80 | 112 | 19.4 | 18 | -1.3 | 45.2 | 43 | -2.0 | 61.0 | 64.6 | 3.2 |
| **PSA (ng/ml)** |  |  |  |  |  |  |  |  |  |  |
| 0<10 | 1,344 | 19.6 | 17 | -0.2 | 90.0 | 94 | 0.3 | 109.6 | 111 | 0.1 |
| 10<20 | 677 | 23.4 | 30 | 1.0 | 71.1 | 57 | -2.1 | 94.5 | 87 | -1.1 |
| 20<50 | 380 | 28.2 | 33 | 1.3 | 51.3 | 50 | -0.3 | 79.4 | 83 | 0.9 |
| ≥50 | 145 | 21.0 | 25 | 2.8 | 22.8 | 24 | 0.8 | 43.7 | 49 | 3.7 |
| **T Stage** |  |  |  |  |  |  |  |  |  |  |
| 1 | 1,625 | 34.2 | 29 | -0.3 | 139.4 | 123 | -1.0 | 173.7 | 152 | -1.3 |
| 2 | 660 | 30.5 | 41 | 1.6 | 66.8 | 74 | 1.1 | 97.3 | 115 | 2.7 |
| 3 | 244 | 23.3 | 27 | 1.5 | 25.6 | 26 | 0.2 | 48.8 | 53 | 1.7 |
| 4 | 17 | 4.1 | 8 | 22.9 | 3.3 | 2 | -7.6 | 7.4 | 10 | 15.3 |
| **Grade Group** |  |  |  |  |  |  |  |  |  |  |
| 1 | 1,126 | 17.6 | 14 | -0.3 | 99.2 | 82 | -1.5 | 116.9 | 96 | -1.9 |
| 2 | 723 | 17.0 | 18 | 0.1 | 56.1 | 62 | 0.8 | 73.1 | 80 | 1.0 |
| 3 | 326 | 15.9 | 23 | 2.2 | 31.8 | 33 | 0.4 | 47.7 | 56 | 2.5 |
| 4 | 170 | 11.0 | 14 | 1.8 | 19.6 | 20 | 0.2 | 30.6 | 34 | 2.0 |
| 5 | 201 | 30.6 | 36 | 2.7 | 28.5 | 28 | -0.2 | 59.0 | 64 | 2.5 |
| **Primary Treatment** |  |  |  |  |  |  |  |  |  |  |
| Conservative Management | 538 | 18.9 | 12 | -1.3 | 69.7 | 66 | -0.7 | 88.6 | 78 | -2.0 |
| RT/RP | 1,836 | 35.6 | 60 | 1.3 | 133.1 | 123 | -0.6 | 168.7 | 183 | 0.8 |
| Hormone Monotherapy | 164 | 37.6 | 33 | -2.8 | 32.2 | 36 | 2.3 | 69.9 | 69 | -0.5 |
| **Ethnicity** |  |  |  |  |  |  |  |  |  |  |
| Chinese | 2,155 | 77.4 | 84 | 0.3 | 205.1 | 194 | -0.5 | 282.5 | 278 | -0.2 |
| Other | 391 | 14.7 | 21 | 1.6 | 30.1 | 31 | 0.2 | 44.8 | 52 | 1.8 |

**Table J** Calibration between observed and predicted prostate cancer specific deaths, non-prostate cancer deaths and overall deaths at 10 years for sub-groups within the Singapore validation cohort using the extended model including PPC.

**7. Model comparison to existing models amongst only men on AS/RT/RP**

|  | **UK** |  |  | **Singapore** | |  |
| --- | --- | --- | --- | --- | --- | --- |
| **PCSM** | C-index | 95%CI | p | C-index | 95%CI | p |
| PREDICT | 0.759 | 0.706-0.812 | - | 0.814 | 0.772-0.856 | - |
| EAU | 0.608 | 0.549-0.665 | <0.001 | 0.756 | 0.716-0.796 | 0.052 |
| NCCN | 0.640 | 0.579-0.701 | <0.001 | 0.785 | 0.733-0.837 | 0.395 |
| CAPRA | 0.667 | 0.607-0.727 | 0.024 | 0.797 | 0.748-0.846 | 0.607 |
| **NPCM** |  |  |  |  |  |  |
| PREDICT | 0.719 | 0.692-0.746 | - | 0.722 | 0.684-0.760 | - |
| EAU | 0.557 | 0.529-0.585 | <0.001 | 0.564 | 0.521-0.607 | <0.001 |
| NCCN | 0.565 | 0.536-0.594 | <0.001 | 0.557 | 0.513-0.601 | <0.001 |
| CAPRA | 0.572 | 0.543-0.601 | <0.001 | 0.575 | 0.530-0.620 | <0.001 |
| **Overall** |  |  |  |  |  |  |
| PREDICT | 0.721 | 0.698-0.744 | - | 0.733 | 0.700-0.766 | - |
| EAU | 0.567 | 0.542-0.592 | <0.001 | 0.613 | 0.578-0.648 | <0.001 |
| NCCN | 0.579 | 0.553-0.605 | <0.001 | 0.615 | 0.577-0.653 | <0.001 |
| CAPRA | 0.590 | 0.564-0.616 | <0.001 | 0.632 | 0.594-0.670 | <0.001 |

**Table K** Comparison of the model to existing models across 15 years within the UK and Singapore validation cohort, excluding all men managed with primary hormone monotherapy. In the Singapore validation cohort, the extended model including the biopsy parameter is used.
PCSM = Prostate cancer specific mortality NPCM = Non prostate cancer mortality
